# Taz protects hematopoietic stem cells from an aging-dependent decrease in PU.1 activity

**DOI:** 10.1038/s41467-022-32970-1

**Published:** 2022-09-03

**Authors:** Kyung Mok Kim, Anna Mura-Meszaros, Marie Tollot, Murali Shyam Krishnan, Marco Gründl, Laura Neubert, Marco Groth, Alejo Rodriguez-Fraticelli, Arthur Flohr Svendsen, Stefano Campaner, Nico Andreas, Thomas Kamradt, Steve Hoffmann, Fernando D. Camargo, Florian H. Heidel, Leonid V. Bystrykh, Gerald de Haan, Björn von Eyss

**Affiliations:** 1 Transcriptional Control of Tissue Homeostasis Lab, Leibniz Institute on Aging, Fritz Lipmann Institute e.V., Beutenbergstr. 11, 07745 Jena, Germany; 2 Leibniz Institute on Aging, Fritz Lipmann Institute e.V., Beutenbergstr. 11, 07745 Jena, Germany; 3 Institute for Research in Biomedicine (IRB Barcelona), Barcelona Institute of Science and Technology (BIST), Baldiri Reixac 10, 08028 Barcelona, Spain; 4 Stem Cell Program, Boston Children’s Hospital, 300 Longwood Avenue, Boston, MA 02115 USA; 5 Department of Stem Cell and Regenerative Biology, Harvard University, 7 Divinity Avenue, Cambridge, MA 02138 USA; 6 Laboratory of Ageing Biology and Stem Cells, European Research Institute for the Biology of Ageing (ERIBA), University Medical Center Groningen (UMCG), University of Groningen, Antonius Deusinglaan 1, 9700 AV Groningen, The Netherlands; 7 Center for Genomic Science of IIT@SEMM, Fondazione Istituto Italiano di Tecnologia (IIT), Via Adamello 16, 20139 Milan, Italy; 8 Institute of Immunology, Jena University Hospital, Am Leutragraben 3, 07743 Jena, Germany; 9 Internal Medicine II, Hematology and Oncology, Jena University Hospital, Am Klinikum 1, 07747 Jena, Germany; 10 Innere Medizin C, Universitätsmedizin Greifswald, Sauerbruchstrasse, 17475 Greifswald, Germany; 11 Sanquin Research, and Landsteiner Laboratory, Academic Medical Center, University of Amsterdam, Amsterdam, The Netherlands

**Keywords:** Haematopoietic stem cells, Transcription

## Abstract

Specific functions of the immune system are essential to protect us from infections caused by pathogens such as viruses and bacteria. However, as we age, the immune system shows a functional decline that can be attributed in large part to age-associated defects in hematopoietic stem cells (HSCs)—the cells at the apex of the immune cell hierarchy. Here, we find that the Hippo pathway coactivator TAZ is potently induced in old HSCs and protects these cells from functional decline. We identify *Clca3a1* as a TAZ-induced gene that allows us to trace TAZ activity in vivo. Using CLCA3A1 as a marker, we can isolate “young-like” HSCs from old mice. Mechanistically, Taz acts as coactivator of PU.1 and to some extent counteracts the gradual loss of PU.1 expression during HSC aging. Our work thus uncovers an essential role for Taz in a previously undescribed fail-safe mechanism in aging HSCs.

## Abstract (web summary)

Immune system function declines with age, a consequence of defects in hematopoietic stem cells (HSCs). Here the authors show that TAZ buffers age-related loss of PU.1 activity to maintain HSC functionality and identify the surface protein Clca3a1 as a marker of “young-like” HSCs, even in old mice.

## Introduction

During aging, hematopoietic stem cells (HSCs) undergo a continuous loss of function accompanied by a reduced regenerative potential^1–3^. Another salient feature of hematopoiesis in the elderly is the decreased production of lymphoid cells, which may contribute to the impaired ability of old individuals to overcome infections and produce an efficient immune response after vaccination^4^. There is ample evidence that these deficiencies are to a large extent due to defects in the hematopoietic stem and progenitor cell (HSPC) compartment. Paradoxically, aging leads to an increased number of immunophenotypic HSCs that exhibit a myeloid bias and poorly reconstitute the blood system after transplantation.

Multiple cell-intrinsic defects contribute to the inferior fitness of old HSC (oHSC), such as altered polarity^5^, decreased autophagy^6^ or epigenomic changes^7^ but the oHSC niche also negatively influences their functionality^8^.

YAP and TAZ are homologous transcriptional coactivators whose activity is restrained by the Hippo signaling pathway^9^. When this pathway is switched off, YAP/TAZ can enter the nucleus and induce gene transcription, mainly by interacting with transcription factors (TFs) of the TEAD family^10^.

Even though altered YAP/TAZ activity can be found in pathological conditions such as cancer^11^, recent studies investigating their physiological role^12^ showed that they are essential to induce regeneration in various tissues, e.g., intestine or liver^13^. YAP/TAZ can reprogram cells ex vivo and in vivo toward a more primitive state which may allow them to better cope with stress during tissue damage^14,15^. *Yap1* or *Wwtr1* (gene encoding TAZ) deletion under homeostatic conditions often leads to a weak or no phenotype^14^, similar to when they are deleted in young HSCs (yHSCs)^16^. However, when cells are confronted with molecular damages, YAP/TAZ are urgently needed to induce a regenerative response^14^.

Given their striking role in regeneration, we sought to investigate whether YAP/TAZ are required to cope with cellular stress and declining regenerative potential in aging HSCs. Here we show that the Hippo pathway coactivator TAZ is highly induced in old HSCs and is critical to maintain a certain level of functionality of these cells. We show that the TAZ-induced gene *Clca3a1* is a proxy for TAZ activity in vivo and can be used to isolate “young-like” HSCs from old mice. In addition, we provide evidence that TAZ acts as coactivator of the hematopoietic transcription factor PU.1 that counterbalances the age-related loss of PU.1 activity in old HSC. With this work, we bring to light an essential and previously undescribed role for TAZ in a fail-safe mechanism preserving HSC function during aging.

## Results

### TAZ is potently induced in oHSCs

**Fig. 1:**
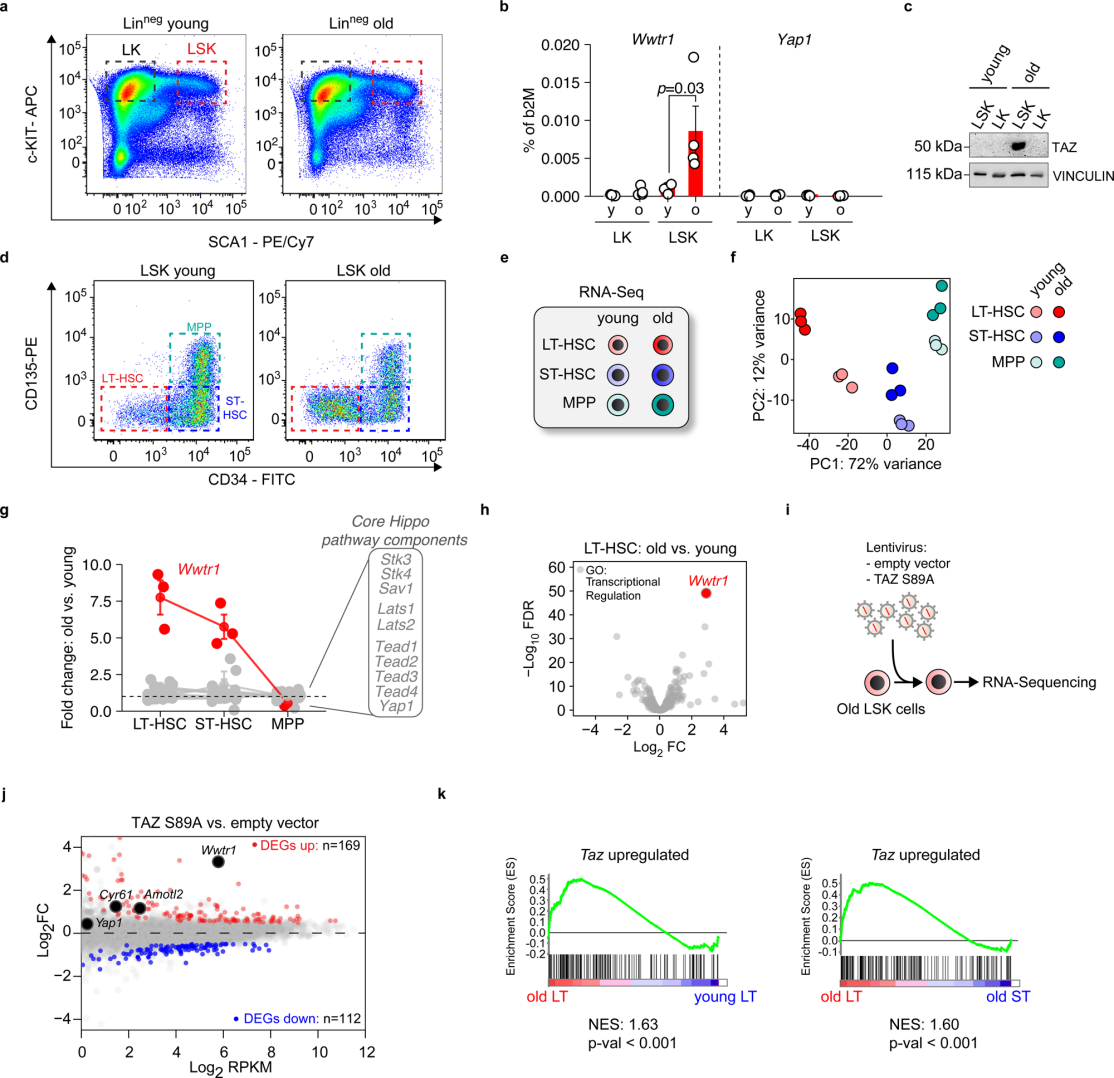
TAZ (*Wwtr1*) is strongly induced in oLT-HSCs. **a** Representative flow cytometry plots of LK (Lineage^neg^, Sca-1^neg^, c-Kit^pos^) and LSK (Lineage^neg^, Sca-1^pos^, c-Kit^pos^) cells of young and old mice. **b** qRT-PCRs of *Wwtr1* and *Yap1* expression in LK and LSK isolated from young and old mice (*n* = 4 per age group, one-way ANOVA with Tukey HSD post hoc test). **c** Immunoblot analysis of TAZ in LK and LSK of young and old mice. **d** Representative flow cytometry plots of LT-HSCs, ST-HSCs, and MPP in the LSK compartment of young and old mice. **e** Overview of the samples that were analyzed by RNA-Seq (*n* = 3 per group). **f** Principal component analysis of the RNA-Seq of the indicated populations (*n* = 3 per group). **g** Summary of gene expression changes (old vs. young) of Hippo pathway core components. **h** Volcano plot encompassing all transcriptional regulators (KEGG database) that were analyzed in the LT-HSC dataset old vs. young. FDR = false discovery rate; FC = fold change. **I** Experimental strategy to identify TAZ-induced genes in oLSK. **j** MA plot of the TAZ S89A overexpression RNA-Seq (*n* = 3 per condition). Significantly up- and downregulated genes (FDR < 0.01) are colored in red and blue, respectively. DEG = differentially expressed genes. **k** Gene set enrichment analysis for the given comparisons applying a TAZ-induced set of 169 genes. NES = normalized enrichment score. Data in **b**, **g** are presented as mean values +/− SEM. Source data are provided as a Source data file.

To investigate the role of *Yap1* and *Taz* (*Wwtr1*) in aging HSPCs, we analyzed their expression in LK (Lin^neg^ Sca-1^neg^ c-Kit^pos^) and LSK (Lin^neg^ Sca-1^pos^ c-Kit^pos^) cells from young and old mice (for exemplary flow cytometry profiles see Supplementary Fig. 1). *Wwtr1* mRNA as well as the Taz Protein were both strikingly induced in old LSK (oLSK), whereas *Yap1* expression remained unchanged (Fig. 1a–c).

To analyze gene expression changes in the LSK compartment in greater detail, we performed RNA-sequencing (RNA-Seq) from long-term HSCs (LT-HSCs: LSK CD34^neg^ CD135^neg^), short-term HSCs (ST-HSCs: LSK CD34^pos^ CD135^neg^) and multipotent progenitors (MPPs: LSK CD34^pos^ CD135^pos^) from young and old mice (Fig. 1d, e). As expected, the number of immunophenotypic LT-HSCs was strongly increased in old mice (Supplementary Fig. 2a).

ST-HSCs and MPPs showed a weaker age-dependent separation in principal component analyses whereas old LT-HSCs (oLT-HSCs) were more clearly separable from young LT-HSCs (yLT-HSCs) and showed the highest number of differentially expressed genes between old and young (Fig. 1f and Supplementary Fig. 2b). Although most Hippo core components showed only weak or no age-dependent changes, *Wwtr1* was strongly increased specifically in LT-HSCs and old human HSPCs (Fig. 1g and Supplementary Fig. 2c). *Yap1*, on the other hand, was barely expressed in LT-HSCs, demonstrating that *Wwtr1* is the predominant transcriptional Hippo effector in these cells (Supplementary Fig. 2d).

Strikingly, *Wwtr1* was the most potently induced transcriptional regulator when comparing oLT-HSCs vs. yLT-HSCs (Fig. 1h).

To identify TAZ-dependent target genes in HSPCs, we lentivirally overexpressed the constitutively active TAZ S89A point mutant in freshly isolated LSKs and subsequently performed RNA-Seq (Fig. 1i, j). A TAZ-induced gene set consisting of 169 genes was then used to probe TAZ activity in HSCs (Fig. 1k and Supplementary Table 1). Gene set enrichment analysis (GSEA) demonstrated that Taz-induced genes were upregulated in oLT-HSCs compared to oST-HSCs and in oLT-HSCs compared to yLT-HSCs (Fig. 1k). These results show that Taz is active in oLT-HSCs and contributes to aging-dependent gene expression changes.

### *Clca3a1* is induced by Taz and marks oLT-HSCs

**Fig. 2:**
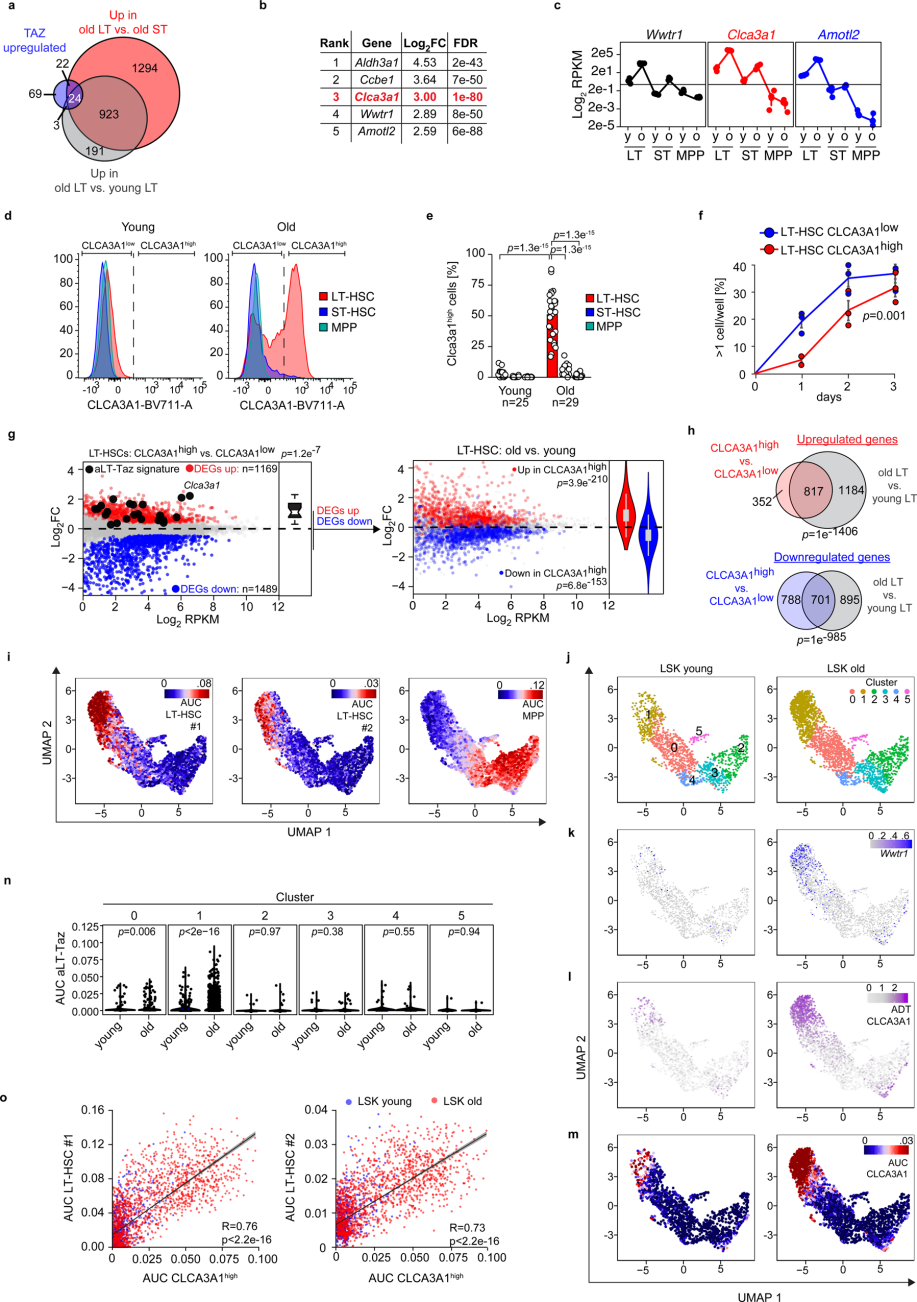
*Clca3a1* is induced by TAZ and marks oLT-HSCs. **a** Overlap among upregulated genes of the indicated RNA-Seq datasets (Log_2_FC > 0.5, FDR < 0.01). **b** Top TAZ-induced LT-HSC-specific genes sorted according to their regulation in old vs. young LT-HSCs. **c** RPKM values (Log_2_ scale) of *Wwtr1*, the TAZ target gene *Amotl2* and *Clca3a1* determined by RNA-Seq (*n* = 3 per age group and population). **d** Representative flow cytometry plots for CLCA31 expression in the given populations from young and old mice. **e** Quantification of samples analyzed in **d** (*n* = 25 for young, *n* = 29 for old mice). **f** Single cell colony-forming assay of isolated CLCA3A1^low^ and CLCA3A1^high^ LT-HSCs. Single cells were seeded and the time to completion of the first cell division was measured (*n* = 3 per group, one-way ANOVA with Tukey HSD post hoc test). **g** Left panel: MA plot of an RNA-Seq comparing CLCA3A1^high^ vs. CLCA3A1^low^ LT-HSCs (*n* = 3 per group, LSK CD34^neg^ CD135^neg^). Significantly up- and downregulated genes (FDR < 0.01) are colored in red and blue, respectively. Right panel: Significantly regulated genes of the CLCA3A1^high^ vs. CLCA3A1^low^ RNA-Seq were colored in the RNA-Seq comparing old vs. young LT-HSCs. Boxplot: bottom/top of box: 25th/75th percentile; upper whisker: min(max(x), Q_3 + 1.5 * IQR), lower whisker: max(min(x), Q_1−1.5 * IQR), center: median. **h** Venn diagram of significantly regulated genes in the given RNA-Seq datasets. The *p*-values for a significant overlap were determined by a hypergeometric test. **i** UMAP dimensionality reduction plots for CITE-Seq data of young and old LSKs. The activity of the indicated gene sets (LT-HSC#1, LT-HSC#2, and MPP) is color-coded based on their AUC. AUC = area under the curve. **j**–**m** UMAP plots for CITE-Seq data of young (left) and old (right) LSKs, respectively. Graph-based clusters (**j**), *Wwtr1* mRNA expression (**k**), CLCA3A1 protein based on ADTs (**l**), and CLCA3A1^high^ gene set activity based on AUCs (**m**) are depicted. **n** Violin plots of TAZ gene set activity (AUC scores) in the different clusters of young and old LSKs, respectively. Cluster 1 contains HSC-like cells, see also (**i**) (two-sided Wilcox test). **o** Scatter plots of CLCA3A1^high^ gene set activity plotted against the activity of two different LT-HSC gene sets #1 and #2, respectively (R = Pearson correlation coefficient, linear regression *t*-test). Data in **c**, **f** are presented as mean values +/− SEM. Source data are provided as a Source data file.

To filter for TAZ-induced genes specific of oLT-HSCs, we intersected the *Taz* overexpression RNA-Seq performed in LSKs with the previous RNA-Seq of young and old HSPCs (Fig. 2a). We obtained a relatively concise set of 24 genes that is also strongly induced in independent datasets (Supplementary Fig. 3a), which we refer to as aged LT-HSC-Taz signature (aLT-Taz signature). In the aLT-Taz signature, *Calcium-activated chloride channel regulator 1* (*Clca3a1*), coding an accessory protein for calcium-activated chloride channels, demonstrated one of the most striking aging-dependent change in LT-HSCs (Fig. 2b). *Clca3a1* expression, just like the TAZ target gene *Amotl2*, strictly followed *Wwtr1* expression in the different HSPC populations suggesting that CLCA3A1 may be used to trace TAZ activity in vivo (Fig. 2c). Remarkably, *Wwtr1*, *Amotl2*, and *Clca3a1* were all part of an aging HSC (aHSC) signature (Supplementary Table 1) generated by a meta-analysis of sixteen transcriptomic datasets of aged HSCs^17^, underlining the robustness of our results.

CLCA3A1 was barely detectable by flow cytometry in young LSK populations but specifically induced in old LT-HSCs (Fig. 2d, e and Supplementary Fig. 1). CLCA3A1^low^ HSCs purified from old animals were less quiescent than yHSCs. It took them significantly shorter to complete their first cell division in vitro^18^ (Fig. 2f), a typical phenotype of oHSCs^19^. CLCA3A1^low^ cells demonstrated a significantly higher Ki67 positivity, potentially because they showed a ~3x higher *Cdk6* expression and thus were more Cdk6-dependent for cell division (Supplementary Fig. 3b–e).

RNA-Seq analysis of sorted CLCA3A1^high^ and CLCA3A1^low^ LT-HSCs (Fig. 2g) identified a high number of DEGs: 1169 upregulated (FDR < 0.01) and 1489 downregulated (FDR < 0.01) in CLCA3A1^high^ vs CLCA3A1^low^.

Notably, the aLT-Taz signature was significantly upregulated (*p* = 1.2e^−7^) in CLCA3A1^high^ LT-HSCs (Fig. 2g), showing that CLCA3A1 is a proxy for TAZ activity.

To analyze the relative contribution of the aging-dependent increase in the frequency of CLCA3A1^high^ cells to the overall transcriptome changes during HSCs aging, we compared the CLCA3A1 RNA-Seq with the old vs. young LT-HSCs RNA-Seq (Fig. 2g). Strikingly, the vast majority of genes that were upregulated in CLCA3A1^high^ cells were also upregulated in old vs. young LT-HSCs, not only in our data but also in an independent RNA-Seq study^7^ comparing old and young LT-HSCs (Supplementary Fig. 3f). The same was true for the downregulated genes. Thus, the up- and downregulated genes in CLCA3A1^high^ cells significantly overlap with the genes up- and downregulated in oLT-HSCs (Fig. 2h). When *Clca3a1* is knocked out in yHSCs in vivo^20^, *Clca3a1* seemed to be required to maintain the HSC pool suggesting that this TAZ-induced gene also has a function in young HSCs (Supplementary Fig. 4). However, we focus here on the upstream coactivator Taz because it regulates a variety of different genes that can affect the ageing phenotype.

### CLCA3A1^high^/TAZ^high^ HSCs mark a specific oHSC subpopulation

To investigate the heterogeneity of Taz-dependent transcription programs in HSPCs at the single-cell level, independently of any surface marker, we analyzed the transcriptome of young LSK (yLSK) and oLSK by *Cellular Indexing of Transcriptomes and Epitopes by Sequencing* (CITE-Seq)^21^. We labeled LSKs with antibody-derived tags (ADTs) for the SLAM marker panel CD150 and CD48 as well as CLCA3A1 to validate our findings with an independent set of HSC surface markers. Based on the activity of specific gene sets (Supplementary Table 1), we were able to identify individual cells with different activities for LT-HSCs, ST-HSCs, and MPPs (Fig. 2i).

Graph-based clustering identified six clusters (clusters 0–5) in the single-cell RNA-Seq (scRNA-Seq) dataset from young and old LSK cells (Fig. 2j). With the help of the SLAM ADTs and gene set activities^22^, we identified cluster 1 as a cluster enriched for LT-HSC (Fig. 2j–m and Supplementary Fig. 3g, h). Since functional studies would be required to demonstrate their functionality as LT-HSCs, we refer to this cluster as ‘HSC-like cluster’. It contained cells expressing high *Taz* on mRNA level (Fig. 2k), high CLCA3A1 on protein level (Fig. 2l) and showing high activity for a CLCA3A1^high^ gene set (Fig. 2m). CLCA3A1^high^ were much more abundant in the aged population (Fig. 2k, l) within the HSC-like cluster’. Only very few scattered yLSKs demonstrated high CLCA3A1 protein level or a high *Clca3a1* gene set activity (Fig. 2l, m and Supplementary Fig. 3i) suggesting that CLCA3A1^high^ cells also exist to a minor extent in young animals. TAZ activity, just like CLCA3A1 activity, was largely limited to the HSC-like cluster and was strongly induced in old cells within this cluster (Fig. 2n).

The CLCA3A1^high^ gene set activity strongly correlated with the activity of two independent LT-HSC-specific gene sets (Fig. 2o), one based on our own RNA-Seq data and one previously published^23^.

In summary, CLCA3A1^high^/TAZ^high^ HSCs represent a specific and highly abundant population within the oHSC compartment. They are characterized by high expression of genes that qualify them as LT-HSCs but are transcriptionally distinct from yHSCs.

### Old CLCA3A1^low^ HSCs show features of “young-like” HSCs

**Fig. 3:**
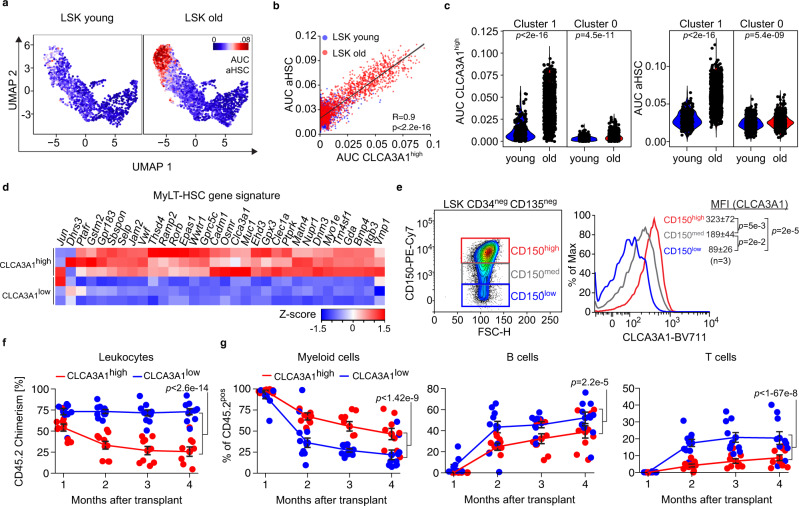
CLCA3A1^high^ HSCs demonstrate features of aged HSCs. **a** UMAP plots for CITE-Seq data of young and old LSKs. The activity of the aged HSC (aHSC) signature is colored based on the gene set activity. **b** Scatter plots of CLCA3A1^high^ gene set activity plotted against the activity of an HSC aging signature from the CITE-Seq dataset (R = Pearson correlation coefficient, linear regression *t*-test). **c** Violin plots of CLCA3A1^high^ (left) and aHSC (right) gene set activity in clusters 0 and 1 from the CITE-Seq (two-sided Wilcox test). **d** Heatmap of Z-score normalized expression values for a gene set for myeloid-biased LT-HSCs (MyLT-HSCs) (*n* = 3 per group). **e** Representative flow cytometry plots for CD150 and CLCA3A1 of oHSCs. The CD150 signal was divided into three different windows (left) and the CLCA3A1 signal was plotted for each window (right). The numbers give the mean fluorescence intensity (MFI) ± 95% confidence interval (one-way ANOVA with Tukey HSD post hoc test). **f** Leukocyte chimerism in the PB over time after competitive transplantation of CLCA3A1^high^ or CLCA3A1^low^ LT-HSCs (*n* = 10 for CLCA3A1^low^, *n* = 9 for CLCA3A1^high^, two independent transplantations, two-way ANOVA with Tukey HSD post hoc test). **g** Percentage of myeloid (CD11b^pos^), B (B220^pos^) and T cells (CD4^pos^ or CD8^pos^) from the donor (CD45.2^pos^) after competitive transplantation of CLCA3A1^high^ or CLCA3A1^low^ LT-HSCs (*n* = 10 for CLCA3A1^low^, *n* = 9 for CLCA3A1^high^, two independent transplantations, two-way ANOVA with Tukey HSD post hoc test). Data in **f**, **g** are presented as mean values +/− SEM. Source data are provided as a Source data file.

To test whether the surface marker CLCA3A1 is sufficient to isolate a pure fraction of oHSCs and “young-like” HSCs from old mice, we compared our CLCA3A1^high^ signature with the aHSC signature. Both signatures nearly perfectly correlated at the single-cell level indicating that CLCA3A1 is an excellent marker to isolate oHSCs (Fig. 3a, b). Remarkably, a substantial number of old cells with low CLCA3A1^high^ gene set activity were present in the HSC-like cluster (Fig. 3c), suggesting that a CLCA3A1^low^ immunophenotype marks individual “young-like” HSCs within the pool of oHSCs.

In accordance with the aged phenotype of CLCA3A1^high^ HSCs, CLCA3A1 induction also correlated with a gene expression profile of myeloid-biased LT-HSCs (MyLT-HSCs)^24^ at the single-cell level (Supplementary Fig. 5a) and in the bulk RNA-Seq dataset (Fig. 3d). Consistently, *Clca3a1* expression clearly correlated with the expression of CD150, a marker of MyLT-HSC, in flow cytometry analyses (Fig. 3e).

Given the fundamental differences between CLCA3A1^low^ and CLCA3A1^high^ HSCs at multiple levels, we next tested whether these two cell populations also differ functionally. To this end, we analyzed their long-term repopulating capacity and their long-term multilineage reconstitution potential in competitive repopulation assays. Two expected hallmarks of oLT-HSCs in this assay are functional decline in long-term repopulation and myeloid skewing of peripheral blood (PB) cells. We transplanted CD45.2-expressing CLCA3A1^high^ or CLCA3A1^ow^ LT-HSCs together with CD45.1/2 competitor cells into lethally irradiated CD45.1 (Ly5.1) recipient mice. PB chimerism was investigated monthly over a period of 4 months. One month post-transplantation, chimerism in the blood of CLCA3A1^high^ recipients was comparable to those who received CLCA3A1^low^ LT-HSCs (Fig. 3f). Over time, however, recipients of CLCA3A1^high^ LT-HSCs showed a gradual decline of donor contribution to the PB (Fig. 3f, g) demonstrating a defect in long-term repopulating activity of the CLCA3A1^high^ population. The frequency of CLCA3A1^low^-derived leukocytes in contrast remained stable compared to the young and age-matched competitor cells (Fig. 3f). In addition, CLCA3A1^high^ LT-HSCs produced significantly more myeloid cells in the PB and fewer B and T cells (Fig. 3g). Four months after transplantation, spleen and bone marrow (BM) chimerism appeared strongly decreased in recipients receiving CLCA3A1^high^ LT-HSCs (Supplementary Fig. 5b, c). Within the donor-derived compartment, Clca3a1^high^- and CLCA3A1^low^-derived cells generated a similar frequency of myeloid progenitors but the CLCA3A1^low^ samples showed a higher frequency of common lymphoid progenitors (CLPs)—a typical characteristic of yHSCs^3^ (Supplementary Fig. 5d). Irrespective of the donor status (CLCA3A1^high^ or CLCA3A1^low^), CLCA3A1 expression was lost in the BM 4 months after transplantation and the two different cell populations showed similar behavior in secondary transplantations.

### Old HSCs induce Taz to keep a minimal regenerative potential

**Fig. 4:**
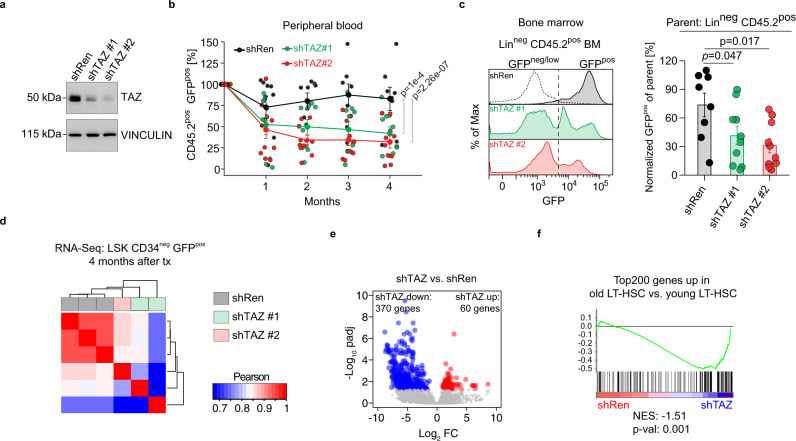
CLCA3A1^high^ HSCs depend on high TAZ expression. **a** Immunoblot of NIH3T3 cells infected with the indicated shRNAs. An shRNA targeting Renilla (shRen) serves as control. The experiment was independently repeated three times. **b** PB analysis after transplantation of CLCA3A1^high^ HSCs transduced with the indicated shRNAs. The GFP^pos^ population in the donor CD45.2^pos^ compartment was analyzed for 4 months post-transplantation (shRen: *n* = 8, shTAZ#1: *n* = 10, shTAZ#2: *n* = 10, three independent transplantations, two-way ANOVA with Tukey HSD post hoc test). **c** Representative GFP flow cytometry plots of Lin^neg^ CD45.2^pos^ BM cells 4 months after transplantation (left). The dotted line indicates the negative control for GFP (gated on CD45.1/2^pos^ supporting cells). Right panel: percentage of GFP^pos^ cells in Lin^neg^ BM cells. The cells were gated on Lin^neg^ CD45.2^pos^ donor cells (shRen: *n* = 8, shTAZ#1: *n* = 10, shTAZ#2: *n* = 10, three independent transplantations, one-way ANOVA with post hoc paired Wilcox test and Benjamini-Hochberg correction). **d** Correlation analysis of RNA-Seq data from LSK CD34^neg^ cells. Old CLCA3A1^high^ HSCs were transduced with the indicated constructs and transplanted. RNA-Seq was performed 4 months after transplantation. **e** Volcano plot of LSK CD34neg cells from (**d**). padj = adjusted *p*-value, FC = fold change. **f** GSEA analysis of shTAZ vs. shRen transduced HSCs using a gene set consisting of the Top200 upregulated genes in old vs. young LT-HSCs. Data in **b**, **c** are presented as mean values +/− SEM. Source data are provided as a Source data file.

Next, we tested whether Taz actively contributes to the functional decline of CLCA3A1^high^ LT-HSCs or whether it gets induced to counteract defects in CLCA3A1^high^ LT-HSCs. Intriguingly, Clusterin (gene name *Clu*) was potently induced in CLCA3A1^high^ LT-HSCs (Supplementary Fig. 6a). Previous studies showed that *Clu* expression is YAP1-dependent and is induced in stressed cells to trigger a regenerative response^25^. To test whether TAZ has a similar function in oHSCs, we depleted TAZ in CLCA3A1^high^ LT-HSCs using two potent shRNAs (shTAZ#1 and shTAZ#2), shTAZ#2 being more potent than shTAZ#1, and a control shRNA (shRen) targeting Renilla (Fig. 4a). We isolated CLCA3A1^high^ LT-HSCs, infected them ex vivo with lentiviral shRNA constructs and transplanted these cells into lethally irradiated recipients. Transplanted CLCA3A1^high^ populations contained ~60–80% infected (GFP-positive) and ~20–40% uninfected (GFP-negative) cells in order to use the GFP-negative fraction as a competitor against the GFP-positive fraction from the same donor and follow the frequency of GFP-positive cells within the CD45.2 donor fraction over time (Supplementary Fig. 6b). In the PB, shTAZ-transduced donor cells rapidly lost the GFP signal demonstrating that uninfected cells outcompeted TAZ-depleted cells whereas shRen cells maintained a stable frequency of GFP-positive cells (Fig. 4b).

TAZ-depleted HSCs showed a tendency to produce increased numbers of myeloid cells and decreased numbers of B and T cells (Supplementary Fig. 6c). The effects appeared subtler when compared to those observed for competitive repopulation, although both *Taz* shRNAs showed similar trends. The inferior fitness of shTAZ progeny was also reflected in the BM where TAZ depletion led to a significantly reduced GFP^pos^ fraction in lineage-negative (Lin^neg^) precursor cells (Fig. 4c). The percentage of CD34^neg^ LSK cells in the GFP-positive compartment, however, remained largely unchanged (Supplementary Fig. 6d) indicating that HSCs were not lost due to Taz depletion. The RNA-Seq analysis of donor-derived LSK CD34^neg^ cells 4 months after transplantation revealed that TAZ-depleted cells were transcriptionally distinct from shRen cells (Fig. 4d, e) and that TAZ depletion led to a downregulation of the top 200 genes that were induced in old LT-HSCs (Fig. 4f). These transcriptional profiling analyses, in conjunction with our transplantation studies of TAZ-depleted HSCs, demonstrate that TAZ sustains a transcriptional program that protects aged HSCs from functional decline.

### Clca3a1^low^ HSCs resemble yHSCs at the chromatin level

**Fig. 5:**
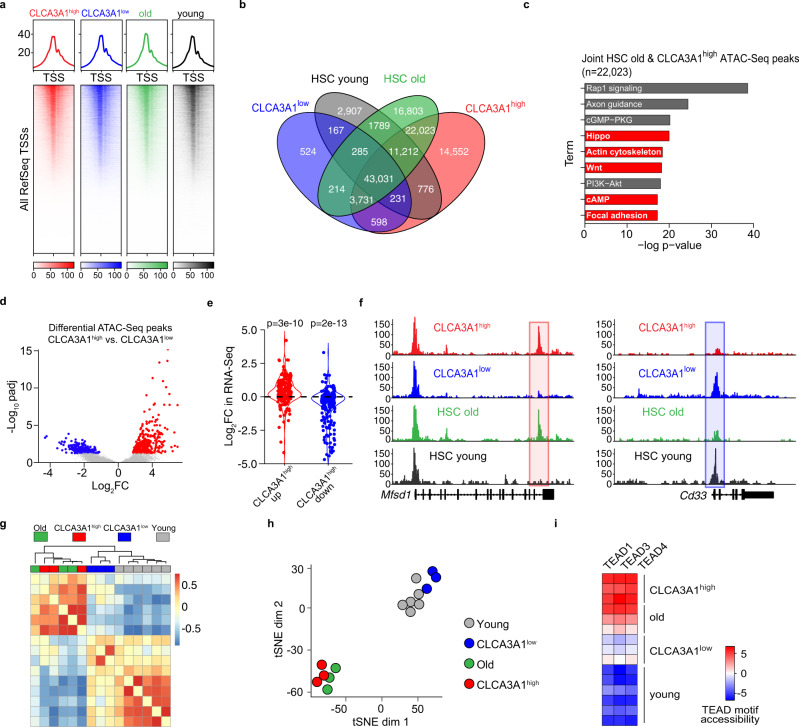
CLCA3A1^low^ HSCs resemble yHSCs on chromatin level. **a** Heatmaps for FAST-ATAC data of CLCA3A1^high^, CLCA3A1^low^ LT-HSCs, oHSCs and yHSCs (*n* = 3 for CLCA3A1^low^, CLCA3A1^high^ and HSC old; *n* = 6 for HSC young). All transcriptional start sites (TSSs) were included and sorted according to the strongest signal. **b** Venn diagram of FAST-ATAC peaks. **c** Gene ontology analysis of FAST-ATAC peaks and their associated genes that are unique to oHSCs and CLCA3A1^high^. The top 10 pathways are depicted. **d** Volcano plot for differential FAST-ATAC peaks comparing Clca3a1^high^ vs. Clca3a1^low^ LT-HSCs. padj = adjusted *p*-value, FC = fold change. **e** Violin plots for the regulation in the RNA-seq dataset of genes that are associated with a differentially accessible FAST-ATAC peak (<25 kb distance from the TSS). Either peaks were used that are significantly more (CLCA3A1^high^ up, red) or less (CLCA3A1^high^ down, blue) accessible in CLCA3A1^high^ vs. CLCA3A1^low^ LT-HSCs (two-sided Wilcox test). **f** Representative genome tracks of the FAST-ATAC experiments of a CLCA3A1^high^ up peak (red box) and a CLCA3A1^high^ down peak (blue box). **g** Clustering of TF motif. accessibility for all motifs in the different samples (*n* = 3 per group). **h** tSNE plot of TF motif accessibility for all motifs in the different samples. **i** TEAD motif accessibility in all peaks of the given samples. Source data are provided as a Source data file.

Given the fundamental changes that occur at the epigenomic level in oHSCs^7^, we analyzed the chromatin changes in CLCA3A1^high^ and CLCA3A1^low^ LT-HSCs. To this end, we profiled the global chromatin accessibility of these cells as well as yHSCs and oHSCs by FAST-ATAC-Seq (Fig. 5a, b)^26^.

We identified 43,031 peaks that were shared between all four samples (Fig. 5b). Strikingly, CLCA3A1^high^ HSCs and oHSCs demonstrated many additional peaks, of these 22,023 were shared between these two samples. The functional GO term analysis of those additional peaks demonstrated a clear enrichment for pathways involved in direct or indirect regulation of TAZ, e.g., Hippo and Wnt signaling pathways (Fig. 5c)^27^. Also, the *Wwtr1* locus itself showed changes in chromatin accessibility specifically in candidate enhancer regions which are hypomethylated in oHSCs^7^ (Supplementary Fig. 7). Differential peak analysis of CLCA3A1^high^ vs. CLCA3A1^low^ HSCs corroborated the trend toward more peaks in CLCA3A1^high^ HSCs (Fig. 5d–f). Increased accessibility also led to greater expression of the associated genes at the mRNA level (Fig. 5e, f), which shows that chromatin changes can impact on gene expression.

To identify changes in TF accessibility indicative of altered TF activity, which could explain the functional differences, we performed a chromVAR analysis^28^. In accordance with our transcriptomic profiling of CLCA3A1^low^ LT-HSCs and yLT-HSCs, CLCA3A1^low^ LT-HSCs clustered together with yLT-HSCs whereas CLCA3A1^high^ LT-HSCs clustered with oLT-HSCs (Fig. 5g, h). Furthermore, the accessibility of the TEAD motifs was much higher in CLCA3A1^high^ LT-HSCs than in yLT-HSCs, indicating that TEAD/TAZ is more active in these cells (Fig. 5i).

Collectively, our data demonstrate that old CLCA3A1^low^ LT-HSCs have lower TEAD/TAZ activity than CLCA3A1^high^ LT-HSCs and resemble yLT-HSCs at the transcriptional, chromatin, and functional level.

### Low PU.1 expression is associated with aging phenotypes

**Fig. 6:**
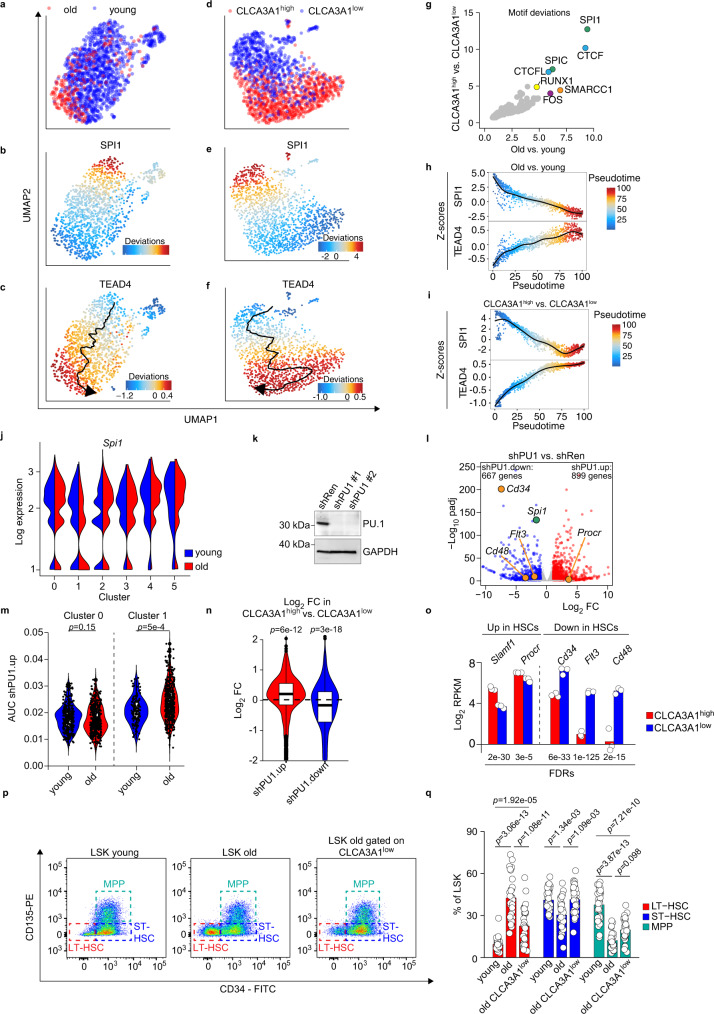
Aging leads to decreased PU.1 activity in CLCA3A1^high^ HSCs. **a**–**f** UMAP plots for scATAC experiments from old vs. young HSCs (**a**–**c**) and Clca3a1^high^ vs. Clca3a1^low^ HSCs (**d**, **e**). Motif accessibility of SPI1 and TEAD4 is color-coded. The pseudotime trajectory is included in **c**, **f**, respectively. **g** Scatter plot that shows the most variable motifs in the given comparisons. **h**, **i** Scatter plot showing the SPI1 and TEAD4 motif accessibility (as Z-score) of individual cells within the pseudotime trajectory for the indicated comparisons. **j** Expression of *Spi1* in the different clusters of the CITE-Seq dataset. **k** Immunoblot for PU.1 in BM-HPC#5 after infection with the indicated shRNAs. Gapdh serves as loading control (experiment repeated once with similar results). The experiment was independently repeated three times. **l** Volcano plot for gene expression changes after PU.1 depletion (shPU1#1 and shPU1#2) in BM-HPC#5 cells. DEGs (padj < 0.05, log_2_FC > 1 or log2FC < (−1), respectively) are colored in blue and red. padj = adjusted *p*-value. **m** Violin plots for the shPU1.up gene set activity in the indicated clusters from the scRNA-Seq dataset of young and old LSK cells (two-sided Wilcox test). **n** Violin plots for the shPU1.up gene set (*n* = 899 genes) and the shPU1.down gene set (*n* = 667 genes) comparing the expression in CLCA3A1^high^ vs. CLCA3A1^low^ LT-HSCs (two-sided Wilcox test). **o** Log_2_ RPKM values of the indicated genes coding for HSC surface markers in CLCA3A1^high^ and CLCA3A1^low^ LT-HSCs, respectively. Data from RNA-Seq comparing CLCA3A1^high^ vs. CLCA3A1^low^ LT-HSCs (*n* = 3 per group, LSK CD34^neg^ CD135^neg^). **p** Representative flow cytometry plots from young and old LSK cells, respectively. In the right panel (LSK old gated on CLCA3A1^low^), the oLSK cells were additionally gated on low CLCA3A1 staining intensity. **q** Barplots summarizing the analysis outlined in **p** (*n* = 33 for young, *n* = 30 for old mice, one-way ANOVA with Tukey HSD post hoc test). Boxplots in **m**, **n**: bottom/top of box: 25th/75th percentile; upper whisker: min(max(x), Q_3 + 1.5 * IQR), lower whisker: max(min(x), Q_1−1.5 * IQR), center: median. Source data are provided as a Source data file.

To understand chromatin accessibility changes in aging HSCs at the single-cell level, we performed single-cell ATAC-Seq (scATAC-Seq) of young and old LT HSCs and of CLCA3A1^high^ and CLCA3A1^low^ LT HSCs (Fig. 6a, d). Analysis of differentially accessible TF motifs showed a striking correlation when comparing changes in old vs. young and CLCA3A1^high^ vs. CLCA3A1^low^ LT-HSCs (Fig. 6g). Here, DNA sequences bound by ETS transcription factors (SPI1 and SPIC) were identified as the most differentially accessible sites in both comparisons as well in our bulk ATAC-Seq data (Fig. 6g and Supplementary Fig. 8a, b). While the accessibility of Spi1 motifs progressively decreased from young to old and from CLCA3A1^low^ to CLCA3A1^high^ LT-HSCs, the accessibility of a Tead motif showed an opposite behavior when we analyzed the behavior of these motifs in pseudotime analyses (Fig. 6h, i). oLT-HSCs are thus a heterogeneous pool of cells where opposed activities of PU.1 and TEAD/TAZ define functionally distinct populations which can be separated based on CLCA3A1. Remarkably, *Spi1* encodes the transcription factor PU.1, a key regulator of HSC function^29^, suggesting that the activity of this critical regulator is decreased during HSC aging. Consistently, *Spi1* expression demonstrated a sharp drop specifically in the HSC-like cluster 1 but not the adjacent cluster 0 in our scRNA-Seq data (Fig. 6j). To investigate whether decreased *Spi1* expression in CLCA3A1^high^ LT-HSCs also affects PU.1 target gene expression, we generated PU.1-dependent gene sets from hematopoietic progenitor cells. For that, we depleted PU.1 in the HSC-like cell line BM-HPC#5^30^ using two potent shRNAs and performed RNA-Seq (Fig. 6k, l). We identified 667 genes which were downregulated (log2FC < (−1), padj<0.05: shPU1.down) and 899 genes that were upregulated (log2FC > 1, padj < 0.05: shPU1.up) upon PU.1 depletion (Fig. 6l). Consistent with low PU.1 expression in CLCA3A1^high^ HSCs, HSC-like cluster 1 contained significantly more cells with high shPU1.up AUC from old vs young, a trend which could not be observed in the adjacent cluster 0 (Fig. 6m). Furthermore, the shPU1.up gene set was significantly upregulated when comparing CLCA3A1^high^ vs. CLCA3A1^low^ LT-HSCs while the shPU1.down gene set showed opposite regulation (Fig. 6n). We noticed that many genes encoding surface markers, commonly used to isolate HSCs (*Cd34*, *Flt3* (CD135), *Cd48*, *Procr* (EPCR)), were affected by PU.1 depletion in BM-HPC#5 and were also downregulated in CLCA3A1^high^ vs. CLCA3A1^low^ LT-HSCs (Fig. 6l, o). Low *Spi1* expression in oHSCs could hence explain the increased frequency of immunophenotypic HSCs commonly observed in aged individuals. We confirmed this by flow cytometry comparing young and old LSK since the frequency of immunophenotypic LT-HSCs in old Clca3a1^low^ LSK resembled that of yLSK cells (Fig. 6p, q).

These data demonstrate that HSC aging is associated with a gradual depletion of PU.1 which correlates with specific features of oHSCs at the transcriptional and immunophenotypic level.

### Decreased *Spi1* expression in yHSC drives features of aging

**Fig. 7:**
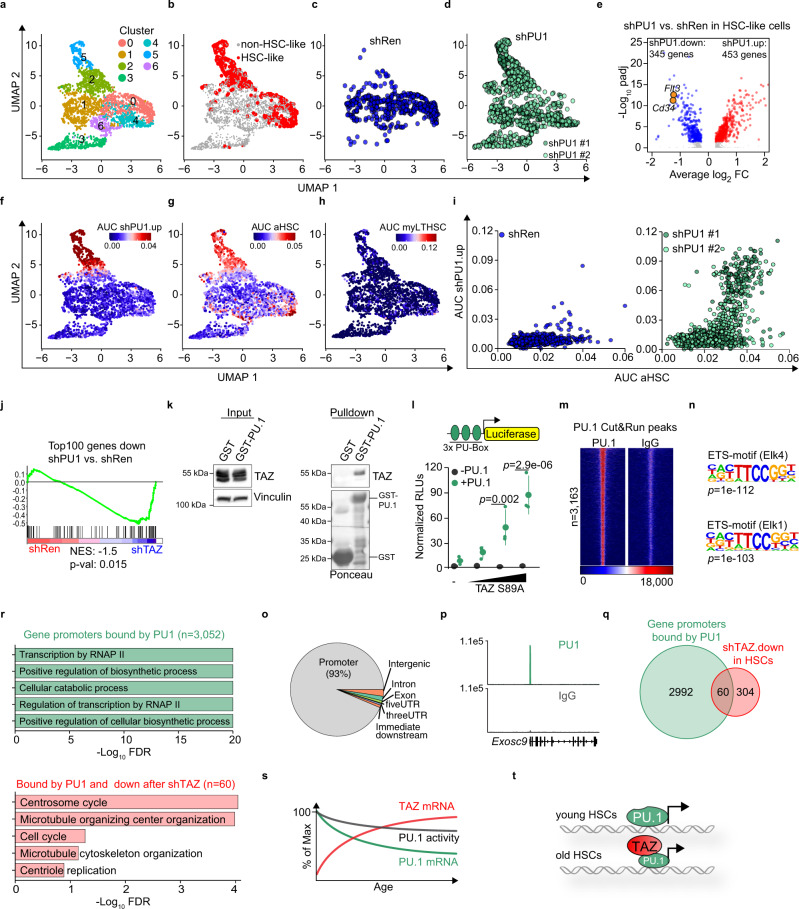
PU.1 depletion in yHSCs leads to “old-like” HSCs. **a**–**d** UMAP plots for CD45.2^pos^ GFP^pos^ LSK cells, isolated 2 months post-transplantation with yHSCs infected with shRen, shPU#1 or shPU#2 lentiviruses. Six clusters (0–6) were identified by graph-based clustering (**a**). HSC-like cells (red) were annotated based on AUC gene set activity for an LT-HSC-specific gene set (**b**) and the corresponding shRNAs are color-coded (**c**, **d**). **e** Volcano plot of differential gene expression in the HSC-like population comparing shRen vs. shPU1 LT-HSCs. **f**–**h** UMAP plots showing the activity of indicated gene sets (color-coded based on the AUC values). **i** Scatter plots showing the aHSC and shPU1.up AUC values of shRen (left) or shPU#1/#2 cells (right) plotted against each other. **j** GSEA analysis of shPU1 vs. shRen transduced HSCs using a gene set consisting of the Top100 genes downregulated by shPU1 in BM-HPC#5 cells. **k** GST pulldowns with cell extracts from 293T cells incubated with either GST or GST-PU-1. Bound TAZ was detected by immunoblot. The experiment was independently repeated three times. **l** Luciferase assay with a reporter construct containing three PU-Boxes. Cells were transfected with increasing amounts of a TAZ S89A construct, either in the absence (−PU.1) or presence (+PU.1) of a PU.1 construct (*n* = 3 independent experiments, one-way ANOVA with Tukey HSD post hoc test). RLUs = relative light units. **m** Heatmap of PU.1 binding at all (*n* = 3163) PU.1 peaks in Cut&Run. IgG serves as negative control. **n** Homer motif analysis, of the most significantly enriched motifs in all PU.1 peaks. **o** Distribution of PU.1 peaks in the genome. **p** Exemplary browser track of PU.1 and IgG Cut&Run signals in the promoter region of *Exosc9*. **q** Venn diagram of gene promoters bound by PU.1 (green) and genes that are downregulated after shTAZ in HSCs (red). **r** Top5 gene ontology terms overrepresented in all PU.1 peaks (*n* = 3523, green) or TAZ-dependent regulated PU.1 peaks (*n* = 60, red). FDR = false discovery rate. **s** Schematic illustrating the changes in TAZ and PU.1 expression and their effect on PU.1 transcriptional activity. **t** Proposed model of how increased TAZ expression in oHSCs maintains PU.1 activity despite reduced PU.1 expression. Data in **k**, **l** are presented as mean values +/− SEM. Source data are provided as a Source data file.

To test whether decreased PU.1 levels in yHSCs is sufficient to induce an “old-like” HSC phenotype, we depleted PU.1 in yHSCs from 14- to 17-week-old by two independent shRNAs. We analyzed the PB and BM only 2 months after transplantation as PU.1-deficient HSCs are lost shortly after transplantation^31^. PU.1-depleted HSCs rapidly lost their ability to contribute to the production of PB cells but had mostly weaker effects on other parameters in BM and PB (Supplementary Fig. 9a–d). Due to the critical role of PU.1 in progenitors downstream of the HSC compartment, we consequently focused on PU.1’s role in HSCs. Therefore, we performed scRNA-Seq of the LSK compartment from control- (shRen) and PU.1-depleted (shPU1 #1 and shPU1 #2) HSPCs 2 months after transplantation (Fig. 7a–d, Supplementary Fig. 9e). Graph-based clustering revealed seven clusters (Fig. 7a). We used a previously defined stringent HSC vs. MPP1 gene set^23^ (LT-HSC #2, Fig. 2i) to identify “HSC-like cells” in our scRNA-Seq data. In this way, we identified two populations consisting mainly of HSC-like cells (Fig. 7b). One population located in clusters 2 and 5 contained almost exclusively PU.1-depleted HSC-like cells while shRen HSC-like cells were mainly found in cluster 0 (Fig. 7c, d). Just as in BM-HPC#5 cells, PU.1 depletion in young HSC-like cells resulted in drastic gene expression changes, with *Cd34* as well as *Flt3* being potently downregulated (Fig. 7e). Consistent with the previously described role of PU.1 in maintaining HSC quiescence, genes induced after PU.1 depletion in HSC-like cells showed highly significant enrichment for E2F-dependent target genes (Supplementary Fig. 9f, g). Noteworthy, *Taz* mRNA was undetectable in the scRNA-Seq dataset, suggesting that PU.1 depletion alone does not lead to *Taz* induction. The shPU1.up gene set showed high activity exclusively in clusters 2 and 5, which contained shPU1 HSC-like cells (Fig. 7f). Strikingly, PU.1 depletion alone was sufficient to induce an “aged-like” transcriptome in young HSC-like cells without inducing a myeloid-biased LT-HSC gene expression program (Fig. 7g, h) suggesting that decreased PU.1 expression in oHSCs does not contribute to this phenotype or that it takes longer to establish. However, the aHSC gene set showed high activity specifically in PU.1-depleted HSC-like cells with low PU.1 activity—equivalent to high sh.PU1.up activity (Fig. 7i).

Given the partially overlapping phenotype that depletion of TAZ and PU.1 had on the regenerative potential of HSCs and their opposite activities in our scATAC analyses, we hypothesized that *Taz* is induced during aging to compensate for the decreased PU.1 expression in oHSCs—possibly because TAZ can act as a coactivator for PU.1. Consistent with this hypothesis, PU.1 and TAZ depletion showed a convergent transcriptional response upon depletion (Fig. 7j). Moreover, PU.1 and TAZ physically interacted in GST pulldown assays and TAZ was able to act as a potent PU.1 coactivator in transient luciferase reporter assays in a strictly PU.1-dependent manner (Fig. 7k, l). Since we were not able to obtain high-quality genomic binding data for PU.1 or TAZ from freshly sorted HSCs, we performed Cut&Run for PU.1 in BM-HPC#5 cells to identify genes that are directly regulated by PU.1. The 3163 PU.1 Cut&Run peaks were highly enriched for ETS-motifs and were almost exclusively located in promoter regions (Fig. 7m–p). To identify direct PU.1 target genes that are induced in a TAZ-dependent manner we intersected the PU.1 peaks with the genes that are significantly downregulated in transplanted old CLCA3A1^high^ LT-HSCs (Fig. 4e) upon TAZ depletion (Fig. 7q). We identified here 60 genes that were potently downregulated (with a median log2 fold change of −4.8) upon TAZ depletion (Supplementary Table 1). Here, the two gene ontology terms centrosome and microtubule organization, which play a critical role in mitosis and symmetric vs. asymmetric cell division, were highly enriched in these 60 genes (Fig. 7r). Thus, our data show that age-dependent loss of PU.1 activity in HSCs is sufficient to induce aging phenotypes and that Taz can counteract them, at least in part, by acting as a PU.1 coactivator for specific genes (Fig. 7s, t).

## Discussion

In this study, we describe a role of the Hippo pathway coactivator TAZ in the context of HSC aging and provide evidence that TAZ is part of a fail-safe mechanism that buffers an age-dependent decline in PU.1 activity specifically in HSCs (Fig. 7s, t). Even though YAP/TAZ do not seem to a play a role in yHSCs under homeostatic conditions^16^, these coactivators are required to maintain quiescence in mice challenged with 5-FU^32^. Therefore, like PU.1^29^, YAP/TAZ are required for the maintenance of HSC quiescence under certain conditions. Low cell cycle activity is normally a prerequisite of an HSC for a competitive performance in transplantation assays. CLCA3A1^low^ HSCs, however, showed a higher cell cycle activity (Supplementary Fig. 3b, c) and yet performed better in competitive transplantation assays than CLCA3A1^high^ HSCs (Fig. 3f). This may be explained by the higher *Cdk6* expression of CLCA3A1^ow^ HSCs, as Cdk6 can induce higher HSC cell cycle activity while maintaining HSC repopulation activity^33^.

Using the TAZ-induced surface marker CLCA3A1 as a proxy for Taz activity, we here demonstrate that one can identify “young-like” HSCs with younger features within the pool of oHSCs—a model that we previously proposed^34^. CLCA3A1^low^ LT-HSCs resemble yHSCs at the immunophenotypic, transcriptional, chromatin and functional level. The aging phenotype of CLCA3A1^high^ HSCs is partly driven by a specific gradual downregulation of PU.1 expression in the HSC compartment which contributes to (at least) two phenotypes commonly observed in oHSCs. First, decreased PU.1 activity in oHSCs impacts on the expression of HSC surface markers (Figs. 6l and 7e), such as *Cd34* and *Flt3*, which can provide an explanation for the immunophenotypic increase in HSCs during aging (Fig. 7o–q). Second, lower PU.1 activity is sufficient to induce an “old-like” HSC expression profile (Fig. 7f–i) and thus could potentially contribute to the concomitant poorer fitness in transplantation assays^3^, independently of its effect on HSC markers. At present, it is unclear whether low PU.1 expression in old HSCs is sufficient to explain some of the defects observed in old HSCs at the functional level. This would need to be investigated using a PU.1 reporter mouse in limiting dilution assays.

It is currently unclear, why HSCs show a gradual loss of *Spi1* expression during aging and how low PU.1 activity is sensed in order to induce TAZ. According to our data, PU.1 depletion itself is not sufficient do induce TAZ suggesting that these two events are not intrinsically coupled. PU.1 functions downstream of the pro-inflammatory cytokine Interleukin1-beta (IL1-ß) to maintain HSC quiescence under inflammatory stress^35^. IL1-ß is induced in the aging BM^36^ suggesting a link between the aging niche and HSC fitness via PU.1. Consistently, we observe that CLCA3A1^high^ HSCs transplanted into young hosts lose their phenotype over time potentially implying an impact of the aging niche whereas systemic factors do not seem to influence HSC fitness^19^. However, we cannot exclude the possibility that the stress of secondary transplantation may lead to reawakening of a previously dormant HSC that becomes dominant after secondary transplantation. As Spi1 binding sites are the most significantly enriched regions exhibiting age-dependent DNA hypermethylation in HSCs^7^, it is likely that a low PU.1 activity state is maintained in oHSCs, at least to some extent, via epigenetic modifications. This should be considered for potential rejuvenation strategies that would aim to restore *Spi1* expression in oHSCs. Such an approach would require to hit a narrow “sweet spot” of *Spi1* expression since *Spi1* overexpression in HSCs also leads to rapid HSC exhaustion^35^.

Myeloid skewing, another hallmark of oHSCs, seems to be rather mildly affected by PU.1 or TAZ depletion since it had only mild or no effect on lymphoid vs. myeloid output and PU.1 depletion failed to induce a myeloid bias expression program (Fig. 7h). We therefore believe that there exist additional TFs whose altered activities contribute to myeloid skewing in CLCA3A1^high^ HSCs—this needs to be investigated in the future.

In conclusion, we uncover a pathway in oHSCs connecting PU.1 and the Hippo pathway coactivator TAZ. Understanding its dysregulation in HSCs aging may facilitate development of therapeutic intervention strategies to improve immune function and a healthier aging in the elderly population.

## Methods

### Reagents

A list of reagents (antibodies, chemicals, cytokines, commercial kits) is provided as Supplementary Table 2.

### Mice

Animal experiments were approved by the state government of Thuringia under the animal experiment license FLI-17-024 and FLI-19-012. Female C57BL/6J or C57BL/6JRj mice used as donors for BM transplantation were obtained from The Jackson Laboratory and the Janvier Labs and recipient female C57BL/6-Ly5.1 mice were provided by the Charles River laboratories. C57BL/6JRj mice were also bred at the Leibniz Insitute on Aging – Fritz Lipmann Institute e.V. (FLI). Young mice were between 3–5 months and old animals were 22–28 months. The mice were kept in individually ventilated cages (IVCs) under Specific Pathogen Free (SPF) conditions with a 12 h/12 h dark/light cycle at a temperature of 20 °C and a relative humidity of 55% according to the directives of the 2010/63/EU and GV SOLAS.

### Cell culture

BM-HPC#5 cells were cultured in IMDM (+GlutaMAX, Thermo Fisher Scientific) supplemented with 5% FBS (Gibco), 1% penicillin-streptomycin (Sigma), 25 ng/mL recombinant mouse SCF (AF-250-03, PeproTech), and 10 ng/mL recombinant IL-6 (78052.1, STEMCELL Technologies).

### Lentiviral constructs

All PCR primers and oligonucleotide sequences can be found in Supplementary Table 3. To generate the Taz lentiviral expression vector pLEGO-iG2-Puro-Flag-Taz, the mouse Wwtr1 cDNA was amplified with primers Wwtr1_Flag_EcoRI/Wwtr1_Stop_NotI from vector pEF-TAZ-N_Flag(S89A) (Addgene #19026) and cloned into the lentiviral expression vector LeGO-iG2-Puro (http://www.lentigo-vectors.de/vectors.htm) between the EcoRI and NotI sites.

To generate mirE-based shRNA lentiviral expression vectors SGE-Ren, SGE-shWwtr1 #1/#2, SGE-shPU.1 #1/#2 DNA or oligos containing the specific shRNA sequences were used as template for PCR with primers mirE_Fw/mirE_Rev. The resulting fragments were then cloned (XhoI/EcoRI) into SGE, a modified version of SGEP (Addgene vector #111170) where the PGK-Puromycin resistance cassette was removed by MluI digestion and re-ligation.

### Lentiviral transduction

LentiX (Takara, Cat.# 632180) cells were used for lentivirus production. Cells were co-transfected with 10 μg psPAX2, 2.5 μg pMD2.G, and 10 μg lentiviral vector using PEI (Polyethylenimine, Sigma). Viral supernatants were harvested 24 h, 48 h, and 72 h after transfection and pooled. HSCs were transduced using RetroNectin (Takara) according to the manufacturer’s specification. Briefly, 96-well flat-bottom polystyrene plates were coated with 100 µl of RetroNectin solution (25 µg/ml in sterile PBS) and kept at 4 °C o/n. The solution was removed and the plates were blocked with 200 µl of 2% BSA (in PBS) for 30 min. The BSA solution was removed, the plates were washed once with PBS and directly used for infection.

250 µl of lentivirus was added per well and centrifuged for 2 h (32 °C, 2000 × *g*). The virus was removed, 500 freshly sorted HSCs were added per well and centrifuged for 10 min (32 °C, 1000 × *g*). The cells were incubated o/n in a cell incubator, washed extensively with 2% FBS (in PBS), and used for transplantation. NIH3T3 cells were a kind gift from Prof. Martin Eilers (Würzburg University, Germany).

### Western blotting

Cells were lysed in RIPA buffer (50 mM Hepes pH 7.9, 140 mM NaCl, 1 mM EDTA, 1% Triton X-100, 0.1% Na-deoxycholate, 0.1% SDS) containing sodium pyrophosphate and protease inhibitor cocktail (Sigma). Lysates were cleared by centrifugation, separated on 8% Bis-Tris gels, and transferred to a PVDF membrane (Millipore). Membranes were blocked with 5% skim milk powder in TBS, probed with primary antibodies diluted in 5% BSA in TBS, and finally incubated with the appropriate horseradish peroxidase-coupled secondary antibodies. Visualization was performed using chemiluminescence HRP substrate (Immobilon Western, Millipore).

### RNA-sequencing

For all RNA-sequencing samples, three biological replicates per condition were analyzed. Total RNA was extracted using RNeasy® Micro Kit (Qiagen) with on-column DNaseI (Qiagen) digestion. RNA integrity (all processed samples had a RIN > 8) was verified with the Agilent Bioanalyzer 2100 automated electrophoresis system (Agilent Technologies). mRNA (at least 10 ng) was isolated using the NEBNext® Poly(A) mRNA Magnetic Isolation Module (NEB) and library preparation was conducted with the NEBNext® Ultra RNA Library Prep Kit for Illumina (NEB) with Dual Index Primers (NEBNext® Multiplex Oligos for Illumina, NEB) following the manufacturer’s description. Cycles for amplification of the cDNA were determined by qRT-PCR. Libraries were quantified with the Agilent 2100 Bioanalyzer automated electrophoresis system (Agilent Technologies) and subjected to 51 bp single-end Illumina Sequencing on a HiSeq 2500 in high-output mode. Reads were extracted in FastQ format using bcl2fastq v1.8.4 (Illumina).

### RNA-sequencing analysis

Adapter removal, size selection (reads > 25 nt) and quality filtering (Phred score > 43) of FASTQ files was performed with cutadapt (http://cutadapt.readthedocs.io/en/stable/guide.html#). Reads were then aligned to the mouse genome (mm10) using bowtie2 (v2.2.9) using default settings. Differential gene expression analysis was performed with edgeR (v3.26.8) using default parameters. PCA analysis was performed using DESeq2 on the 500 most variable genes.

### FAST-ATAC-Seq

The ATAC-Seq experiments were performed as described previously^34^. Three biological replicates per condition were analyzed. Briefly, 5000 cells resuspended in 50 µl of Transposase mixture: 1x Tagment DNA buffer, 0.5% TDE1 both from (Illumina Tagment DNA TDE1 Enzyme and Buffer Kits, 20034197) and 0.01% Digitonin (Sigma-Aldrich, D141-500MG). Cells were incubated for 30 min at 37 °C, 300 rpm, and the reaction was stopped by proceeding with DNA purification using the ChIP DNA clean & Concentrator (Zymo Research). The transposed DNA fragments were preamplified by a first PCR reaction with 5 cycles containing barcoded Nextera PCR primers. The optimal number of cycles was determined by a SybrGreen qPCR reaction containing a 5 µl aliquot from the first PCR. The second PCR was then carried out with 8 cycles and the libraries were purified by AMPure XP beads. Libraries were quantified with the Agilent 2100 Bioanalyzer and sequenced using a NextSeq500 and a 75 cycle High Output v2.5 kit in paired-end mode (40 bp each read). Extraction of FastQ files was done using bcl2fastq v2.20.0.422 (Illumina).

### FAST-ATAC-Seq analysis

For the primary analysis (Quality filtering, adapter trimming, mapping, removal of PCR duplicates) we used a publicly available ATAC-Seq pipeline (https://github.com/nf-core/atacseq). Peak calling was performed with Genrich (https://github.com/jsh58/Genrich) using default parameters and the overlap of the peaks was determined with the help of ChIPpeakAnno (https://www.bioconductor.org/packages/release/bioc/vignettes/ChIPpeakAnno/inst/doc/ChIPpeakAnno.html). Functional GO term annotation of ATAC-Seq peaks was performed using HOMER’s findGO option after annotating the peaks with the closest gene using the annotatePeaks option in HOMER. ChromVAR (https://bioconductor.org/packages/release/bioc/html/chromVAR.html) was used to analyze the chromatin accessibility of transcription factor motifs in peaks. Heatmaps and normalized bigwig files were generated by deepTools (https://deeptools.readthedocs.io/en/develop/).

### Statistics and reproducibility

All statistical analyses were performed in R (v4.1.0). The graphs always display the mean value and the standard error of the mean (SEM) unless stated otherwise. The statistical test performed is always given in the respective Figure legend.

### qRT-PCR

RNA was extracted with peqGOLD TriFast Reagent (Peqlab). First-strand cDNA synthesis was performed using M-MLV Reverse Transcriptase (Promega) and random hexamers (Sigma) according to standard procedures. PCR reaction was performed in technical triplicates using Innumix SybrGreen Mix (Analytik Jena). Gene expression was analyzed with a StepOnePlus™ Real-Time PCR System (Thermo Scientific). The expression values were normalized to *B2m* as housekeeping gene using the ddCt method. The primer sequences are listed in Supplementary Table 3.

### Isolation of primary HSPCs from mouse and flow cytometry

Femurs, tibias, hips, humeri, and spine were isolated from female C57BL/6JRj mice (Janvier Labs) of various ages and crushed in ice-cold FCM buffer (2% FCS, 1 mM EDTA in PBS). Cells were filtered through a 70 μm cell strainer and subjected to c-kit enrichment using an anti-c-Kit-APC antibody (BioLegend), anti-APC magnetic beads (Miltenyi Biotec), and LS MACS columns (Miltenyi Biotec). c-kit enriched cells were then incubated with a mixture of biotin-conjugated lineage antibodies (ThermoFisher Scientific) (against Gr1, TER-119, B220, CD11b, CD3, CD4, and CD8). When required, unconjugated anti-Clca3a1 antibody (DSHB) was also added in this step. Cells were subsequently washed with FCM and stained with fluorophore-conjugated antibodies. If anti-Clca3a1 antibody was added to the lineage antibody mix, anti-syrian hamster IgG2 conjugated to Brilliant Violet 711 dye (BioLegend) was also used in combination with HSC staining. For flow cytometric analysis and subsequent sorting, cells were washed once after staining and resuspended in FCM. The cell suspension was then filtered through (40 µm) and 1 μM SYTOX Blue dead cell stain (Thermo Fisher Scientific) was added. Fluorescence-activated sorting panels were set up using the automatic compensation feature of the FACS Diva software (BD Biosciences) according to the manufacturer’s instructions with unstained cells or Ultracomp eBeadsTM (Thermo Fisher Scientific) as single-stained compensation controls (see the eBeadsTM manual for the protocol). Cells were sorted into BSA-coated 15 mL low-binding Falcon tubes with 0.1% BSA in IMDM using the BD FACSAria Fusion cell sorter (BD Biosciences) and were stored on ice until further processing.

### Single-cell colony assay

Freshly isolated or transduced LSK cells were plated into round-bottom 96-well plate as a single cell per well containing 100 μL of single cell colony formation medium composed of StemSpan^TM^ Serum-free expansion medium (SFEM, #09650, STEMCELL Technologies) supplemented with 1% Penicillin-Streptomycin (Thermo Fisher Scientific) and 10 ng/mL recombinant mouse SCF, TPO, and IL-3 (STEMCELL Technologies) by BD FACSAria Fusion or FACS Melody™. Images of individual wells were taken by the IncuCyte S3 Live-Cell Analysis System (Sartorius) according to the manufacturer’s instructions.

### Culture of primary murine HSPCs

Freshly sorted LSK cells (1–2 × 10^4^ cells/replicate) were seeded into 200 μL of HSPC culture medium composed of StemSpan^TM^ Serum-free expansion medium (SFEM, #09650, STEMCELL Technologies) supplemented with 1% Penicillin-Streptomycin (Thermo Fisher Scientific) and 50 ng/mL recombinant mouse SCF and TPO (STEMCELL Technologies) per well of a 48-well-plate and incubated at 37 °C, with 5% CO_2_ and 95% relative humidity. After 8–24 h of incubation, cells were transduced by concentrated lentivirus in SFEM medium supplemented with 10 ng/mL SCF, TPO, and IL-3 (STEMCELL Technologies). Forty-eight hours post transduction, cells were washed extensively in 0.1% BSA/PBS and single cells were plated in 96-well plates containing HSPC culture medium. For RNA-Sequencing from Taz S89A overexpressing LSK cells, RNA was isolated 3 days after lentiviral transduction.

### Competitive bone marrow transplantation (BMT) assay

BMT assay was carried out according to protocols approved by the state government of Thuringia under the animal experiment license FLI-17-024. To study their ability for multilineage reconstitution in vivo, Clca3a1^high/low^ CD34^neg^ LSK cells were isolated from donor female C57BL/6JRj mice (Janvier Labs) and purified by flow cytometry (BD Biosciences FACSAria Fusion cell sorter). Cells were either used as such for transplantation or were subjected to lentiviral transduction to investigate the effect of Taz depletion. 8–12-week-old C57BL/6-Ly5.1 recipient mice (Charles River) were subjected to whole body irradiation (12 Gy γ-irradiation from a Caesium-137 source) on the day of the transplantation. Unfractionated BM cells of young (<30 weeks) CD45.1/CD45.2 female C57BL/6JRj mice were transplanted as competitor cells together with the donor cells. To this end, 500 Clca3a1^high/low^ HSCs were transplanted per recipient by tail vein injection along with 2 × 10^5^ CD45.1/CD45.2 competitors. The recipient mice received fresh sterile drinking water with 0.01% Enrofloxacin (Baytril®, Bayer, Germany) each day during the first week after transplantation to prevent the risk of bacterial infections.

### Peripheral blood analysis

PB analysis of the recipient mice was performed every 4 weeks post-transplantation until sacrifice. Blood samples were collected from the *Vena facialis* into tubes containing 8 µl of 0.5 M EDTA and stained with fluorophore-conjugated antibodies (BioLegend) against CD45 variants and lineage markers for 30 min on ice (anti-CD45.1-PE, anti-CD45.2-PerCP/Cy5.5, anti-Gr1-PE-Cy7, anti-CD11b-APC-Cy7 or CD11b-FITC, anti-CD4-Alexa Fluor 700, anti-CD8a-APC, anti-B220-Brilliant Violet 605). Red blood cells were then lysed in 1X BD Pharm Lyse lysing solution (BD Biosciences) for 8–10 min. Cells were washed twice in FCM, resuspended in fresh FCM, and filtered through a 40 μm mesh. After addition of 1 μM SYTOX Blue dead cell stain (ThermoFisher Scientific), cells were subjected to flow cytometry analysis using the BD LSR Fortessa (BD Biosciences).

### Bone marrow analysis

Four months after transplantation, recipient mice were sacrificed using CO_2_ and the total BM was isolated by flow cytometry. The BM samples were split between two wells of a 96-well-plate to be stained with two different fluorophore-conjugated antibody cocktails. For the analysis of chimerism and progenitor populations, BM cells were first stained with biotin-conjugated lineage antibodies and anti-Clca3a1 antibody in 50 μL volume of FCM buffer. After washing in FCM, cells were incubated with anti-c-kit-APC, anti-Sca1-PE-Cy7, streptavidin-EFluor450, anti-CD34-FITC, anti-hamster-Brilliant Violet 711, CD45.1-Alexa Fluor 700, CD45.2-PerCP-Cy5.5, CD127-Brilliant Violet 605, and CD16/32-APC-Cy7 (ThermoFisher Scientific and BioLegend). Finally, cells were washed, filtered, stained with SYTOX Blue dead cell stain (Thermo Fisher Scientific) and analyzed using the BD LSR Fortessa (BD Biosciences).

### CITE-Seq of sorted LSK cells

For CITE-Seq, LSK cells were isolated from young and old mice (four per age group) and purified by flow cytometry as described above with some modifications: BM cells were stained simultaneously with antibodies for sorting and CITE-Seq in three steps. BM cells were incubated with an anti-Clca3a1 antibody, then with a lineage mix of biotinylated antibodies together with an anti-syrian hamster PE/Cy7 antibody and finally the cells were stained with streptavidin eF450, anti-c-Kit-APC and anti-Sca1-FITC. At this last step all Total-seq antibodies were added: anti-PE, anti-CD150, anti-CD48+ isotype controls. After sorting in PBS 0.04% BSA, cells were washed, counted with an automated cell counter (EVE, NanoEnTek), and adjusted to a concentration of 1000 cells/μL.

Cells were subjected to the Chromium single cell 3’ assay v3 (10X Genomics) as recommended by the manufacturer. After cDNA amplification, ADT-derived cDNAs and mRNA-derived cDNAs were separated based on their size using 0.6x AMPure XP Beads (Beckman Coulter). The mRNA-derived cDNAs contained in the bead fraction were further processed following the standard 10X Genomics protocol in order to generate single-cell (sc)RNA libraries. The ADT-derived cDNAs contained in the supernatant were further purified as follows: 1.4x beads were added to obtain a final AMPure beads concentration of 2x and samples were incubated 10 min at RT. Supernatant was discarded and beads were washed with Ethanol 80%, air-dried and resuspended in 50 µL water. The same procedure was repeated once. Beads were washed twice with Ethanol 80% and air-dried. The purified ADTs were subsequently eluted in 45 μL water at RT for 5 min. To generate the ADT sequencing library, 45 μL ADTs were used as template in a 100 µL PCR reaction with the NEBNext® High Fidelity Master Mix (NEB), a Truseq small RNA RPIx (containing i7 index) primer and the 10X Genomics SI-PCR primer (see primer table). The PCR products were incubated with 1.6x AMPure beads at RT for 5 min. After washing the beads twice with Ethanol 80%, the ADT library was eluted in 30 μL water. The libraries were quantified using an Agilent Tapestation 4200. The scRNA-Seq libraries and the ADT libraries were sequenced together on an Illumina NextSeq500 platform using a 75 cycle High Output v2.5 kit. The following sequencing cycles were performed: R1 (10x barcode + UMI): 26 bp; R2 (cDNA): 53 bp; i7 index: 8 bp. Extraction of FastQ files was done using bcl2fastq v2.20.0.422 (Illumina).

### CITE-Seq analysis

FASTQ files from the sequencing runs were analyzed using the 10X Genomics Cell Ranger (v3.1.0) software. Briefly, the *cellranger count* function was used to count the UMIs/cell and the datasets were subsequently normalized by *cellranger aggr*. The resulting count matrix was subsequently analyzed by Seurat (4.0.3). Only cells with less than 10% mitochondrial counts and more than 1500 unique features were kept for further analysis. In order to perform a comparative analysis of old vs. young LSK cells, the data were normalized by SCTransform and an integrated analysis using the FindIntegrationAnchors function (with dims = 30) was performed.

The activity of gene sets was analyzed by AUCell^31^ and added to the Seurat object. The UMIs of the ADTs were counted and mapped using the CITE-seq-Count tool (https://hoohm.github.io/CITE-seq-Count/) using the following options: CITE-seq-Count -R1 R1.fastqgz -R2 R2.fastq.gz -cbf 1 -cbl 16 -umif 17 -umil 26 -cells # of cellranger output. All ADT data were log-transformed.

### scATAC-Seq

The data were obtained from four mice, with each mouse contributing the same number of cells to the sample. 10,000 cells of Clca1^high^ and Clca1^low^ LT-HSCs were sorted into round-bottom 96-well plate containing 0.04% BSA/PBS. For nuclei isolation, cells were lysed with chilled lysis buffer (10 mM Tris-HCl pH 7.4, 10 mM NaCl, 3 mM MgCl_2_, 0.1% Tween 20, 0.1%Nonidet P40, 0.01% Digitonin, 1% BSA) on ice for 3 min. Cells were washed with wash buffer (10 mM Tris-HCl pH 7.4, 10 mM NaCl, 3 mM MgCl_2_, 0.1% Tween 20, 1% BSA). Supernatant was discarded and cells were resuspended in 1X Nuclei Buffer (10X Genomics, PN-2000153/2000207). Nuclei suspension was directly subjected into library preparation using Chromium Next GEM Single Cell ATAC kit v1.1 (10X Genomics) according to the manufacturer’s description. Libraries were quantified with the Agilent 2100 Bioanalyzer. Sequencing was done using a NextSeq500 in combination with a 75 cycle High Output v2.5 kit. The following sequencing cycles were performed: R1 (sequencing of interest: 33 bp; R2 (sequencing of interest): 33 bp; i7 index (sample index): 8 bp; i5 index (10X barcode): 16 bp. Extraction of FastQ files was done using bcl2fastq v2.20.0.422 (Illumina).

### scATAC-Seq analysis

FASTQ files were analyzed by the 10X Genomics cellranger-atac pipeline (v1.2.0). The transcription factor motif accessibility is taken from the cellranger output. ChromVAR was used to cluster the cells based on their accessibility in peaks for all motifs from the JASPAR2016 database comprising 386 transcription factor binding motifs.

### Cut&Run

Briefly, for each CUT and RUN reaction 200,000 BM-HPC#5 cells were washed, resuspended in 100 μl wash buffer (20 mM HEPES, pH 7.5, 150 mM NaCl, 0.5 mM Spermidine) and bound to 10 μl activated concanavalin A magnetic beads for 10 min at room temperature.

Bead-bound cells were then incubated in 100 μl antibody buffer (wash buffer + 0.01% digitonine and 2 mM EDTA) with the desired antibody (1:100) overnight at 4 °C. As negative control an IgG rabbit antibody was used. Beads were washed in digitonin wash buffer (wash buffer + 0.01% digitonin) and incubated 1 h at 4 °C with 1 μg/mL protein A/G Micrococcal Nuclease fusion protein (pA/G MNase). After 3 washing steps in digitonin wash buffer, beads were rinsed with Low salt buffer (20 mM HEPES, pH 7.5, 0.5 mM Spermidine, 0.01% digitonine) and placed in 200 µl incubation buffer (20 mM HEPES, pH 7.5, 10 mM CaCl_2_, 0.01% digitonin) at 0 °C to initiate cleavage. After 30 min, reactions were stopped by adding 200 µl STOP buffer (170 mM NaCl, 20 mM EGTA, 0.01% digitonin, 50 µg/ml RNAse A) and the samples were incubated 30 min at 37 °C to digest the RNA and release the DNA fragments.

The samples were then treated with proteinase K for 1 h at 50 °C and the DNA was purified using Phenol/Chloroform/Isoamyl alcohol. After precipitation with glycogen and Ethanol, the DNA pellet was resuspended in 0.1 X TE and used for DNA library generation with the NEBNext® Ultra™ II DNA Library Prep Kit for Illumina® (New England Biolabs) according to the manufacturer’s recommendations. Adapter ligation was performed with 1:25 diluted adapter and 15 cycles were used for library amplification.

### Luciferase assay

LentiX (293T) cells were transfected with a pGL4-20 luc2/Puro (Promega) vectors containing a PU-box reporter construct using PEI reagent. pCDNA3 and pCDNA3-HA-PU.1 were co-transfected together with increasing amounts of pLeGO-iG2-Puro and pLEGO-iG2-Puro-Flag-TAZ(S89A). Forty-eight hours post-transfection, cells were lysed with passive lysis buffer (Promega) and luciferase activity was measured in a Luminometer (Berthold Technologies). Equal amounts of a CMV-Renilla construct were always co-transfected and firefly luciferase light units were normalized to Renilla luciferase activity.

### Reporting summary

Further information on research design is available in the Nature Research Reporting Summary linked to this article.

## Supplementary information

Supplementary Information

Reporting Summary

## Data Availability

The Next-generation sequencing data generated in this study have been deposited in the GEO database under accession code GSE157464. Source data are provided with this paper.

## Source data

Source Data
