# Supplementary material for: Taz protects hematopoietic stem cells from an aging-dependent decrease in PU.1 activity
Source: Nat Commun. 2022 Sep 3;13:5187. doi: 10.1038/s41467-022-32970-1 (PMC9440927; doi:10.1038/s41467-022-32970-1)
Supplement: Supplementary file 1 — Supplementary Information [file 41467_2022_32970_MOESM1_ESM.pdf]

a

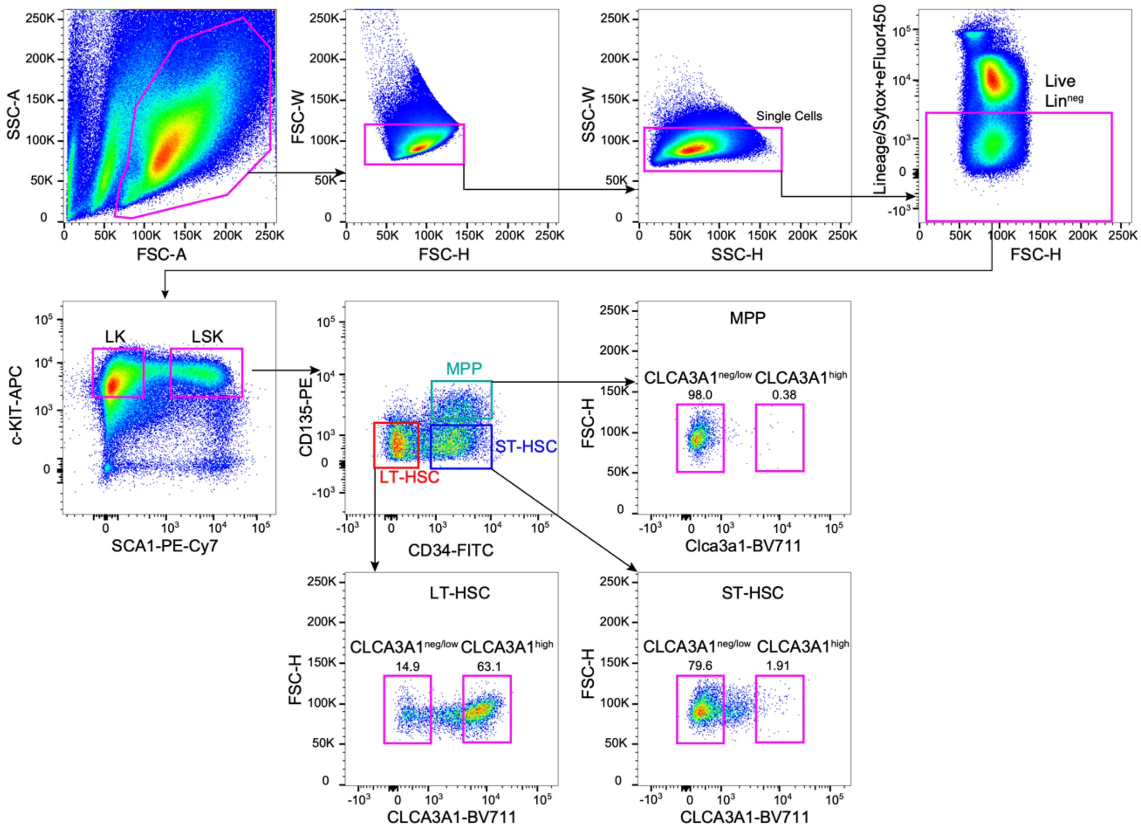
SSC-A
FSC-A
FSC-W
FSC-H
SSC-W
SSC-H
Single Cells
Lineage/Sytox+eFluor450
Live
Lin^neg
c-KIT-APC
SCA1-PE-Cy7
LK
LSK
CD135-PE
CD34-FITC
MPP
ST-HSC
LT-HSC
MPP
CLCA3A1^neg/low CLCA3A1^high
98.0
0.38
Clca3a1-BV711
LT-HSC
CLCA3A1^neg/low CLCA3A1^high
14.9
63.1
CLCA3A1-BV711
ST-HSC
CLCA3A1^neg/low CLCA3A1^high
79.6
1.91
CLCA3A1-BV711

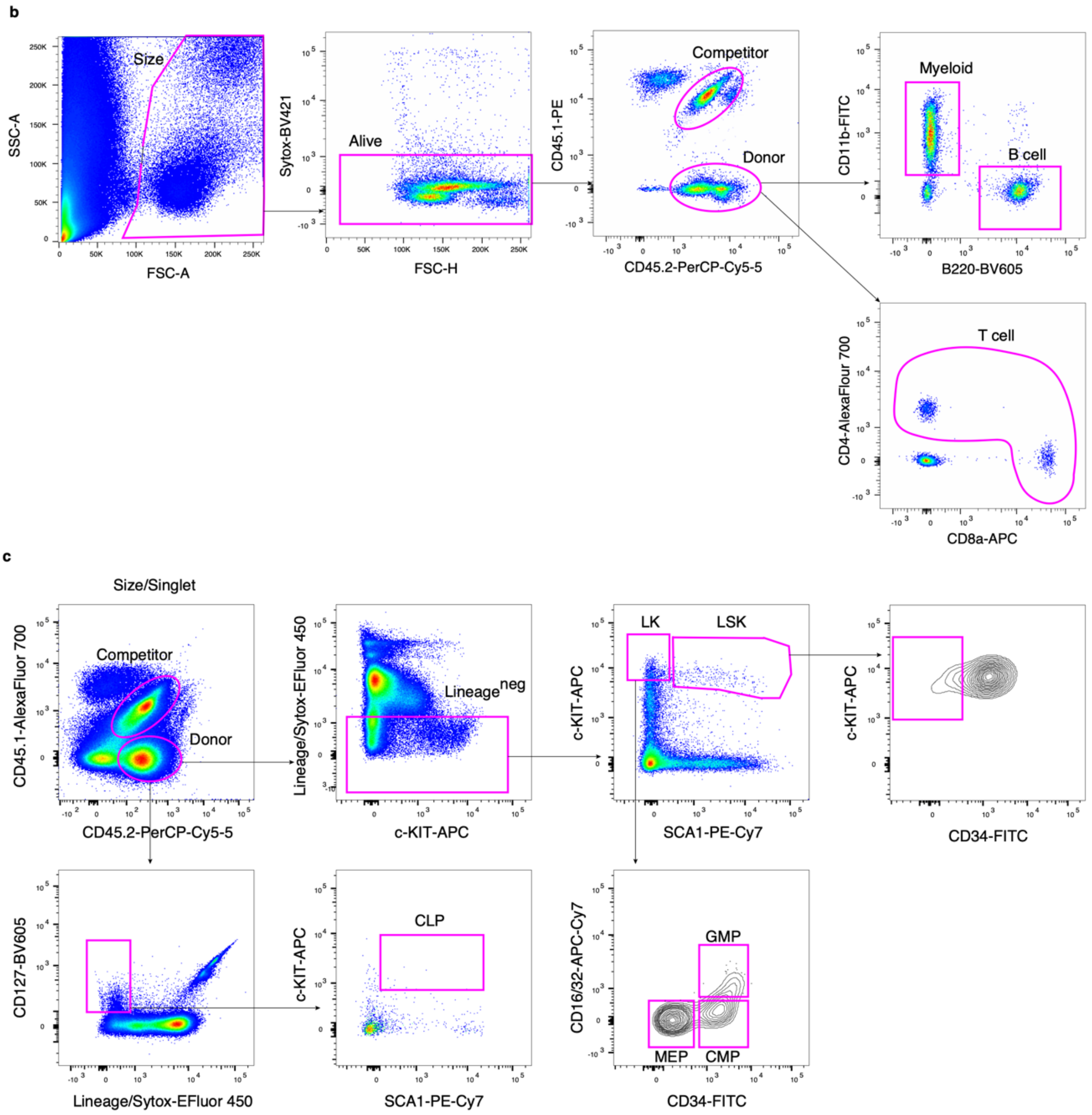
b
Size
SSC-A
FSC-A
Alive
Sytox-BV421
FSC-H
Competitor
Donor
CD45.1-PE
CD45.2-PerCP-Cy5-5
Myeloid
B cell
CD11b-FITC
B220-BV605
T cell
CD4-AlexaFlour 700
CD8a-APC
c
Size/Singlet
Competitor
Donor
CD45.1-AlexaFluor 700
CD45.2-PerCP-Cy5-5
Lineage^neg
Lineage/Sytox-EFluor 450
c-KIT-APC
LK
LSK
c-KIT-APC
SCA1-PE-Cy7
c-KIT-APC
CD34-FITC
CD127-BV605
Lineage/Sytox-EFluor 450
CLP
c-KIT-APC
SCA1-PE-Cy7
GMP
MEP
CMP
CD16/32-APC-Cy7
CD34-FITC

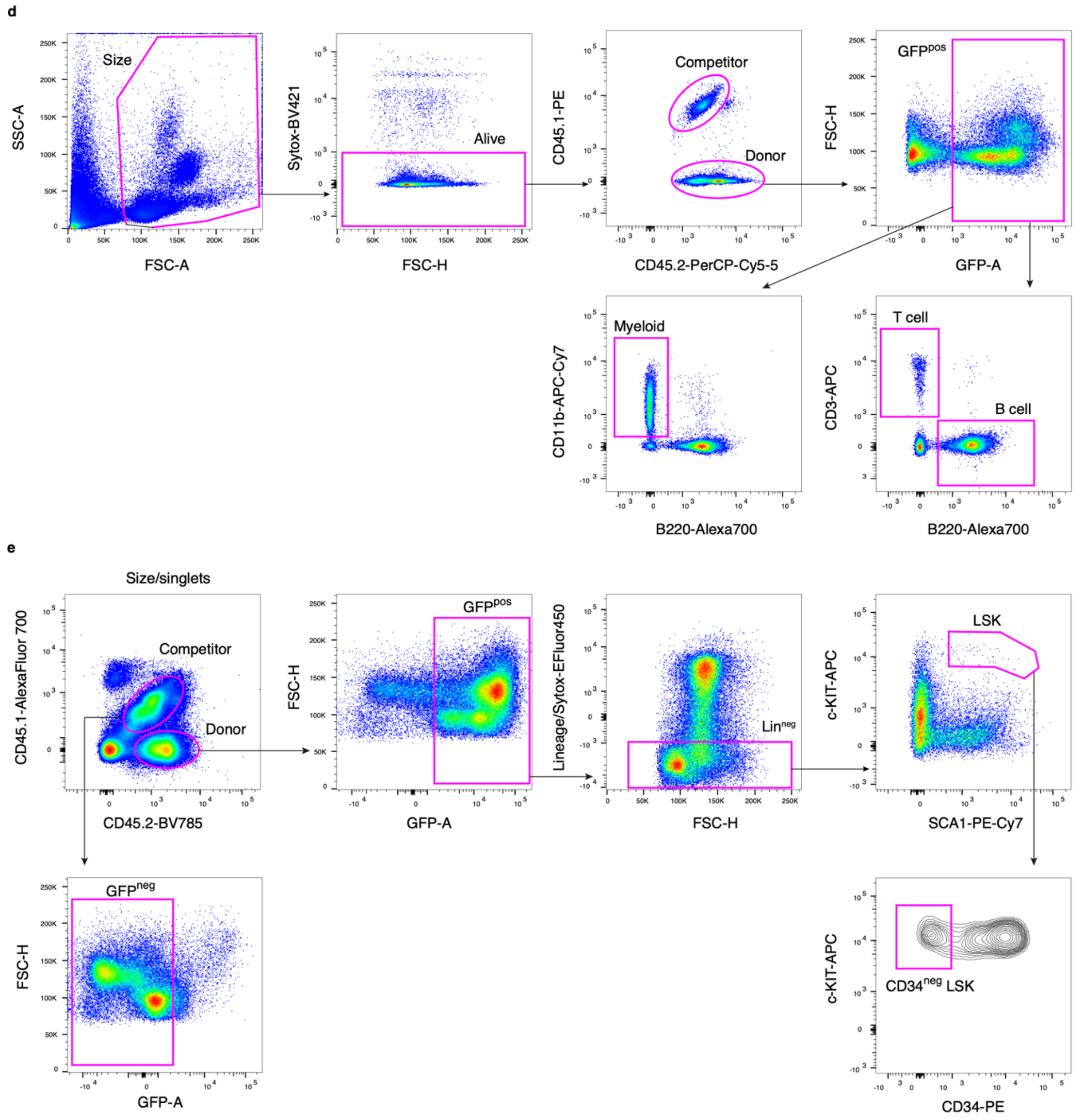

Supplementary Fig. 1: Representative flow cytometry plots of all analyses performed in this study.

a. LT-HSCs, ST-HSCs, MPPs, Clca3a1^{high} and Clca3a1^{low} LT-HSCs in bone marrow: Flow cytometry plots of c-Kit enriched bone marrow from an old mouse that illustrates the stainings of the indicated cell populations and the gating strategy used for cell sorting.

b. Peripheral blood analysis of a recipient that illustrate the stainings of the indicated cell populations and the gating strategy used for analysis. This analysis was performed four months after transplantation.

c. Bone marrow analysis of a recipient that illustrate the stainings of the indicated hematopoietic progenitor cell populations and the gating strategy used for analysis. This analysis was performed four months after transplantation.

d. Peripheral blood analysis of a recipient reveicing HSCs transduced with a lentivirus expressing GFP along with the shRNA. The stainings of the indicated cell populations and the gating strategy used for analysis are indicated. This analysis was performed four months after transplantation.

e. Bone marrow analysis of a recipient reveicing HSCs transduced with a lentivirus expressing GFP along with the shRNA. The stainings of the indicated hematopoietic progenitor cell populations and the gating strategy used for analysis are indicated. This analysis was performed four months after transplantation. Source data are provided as a Source Data file.

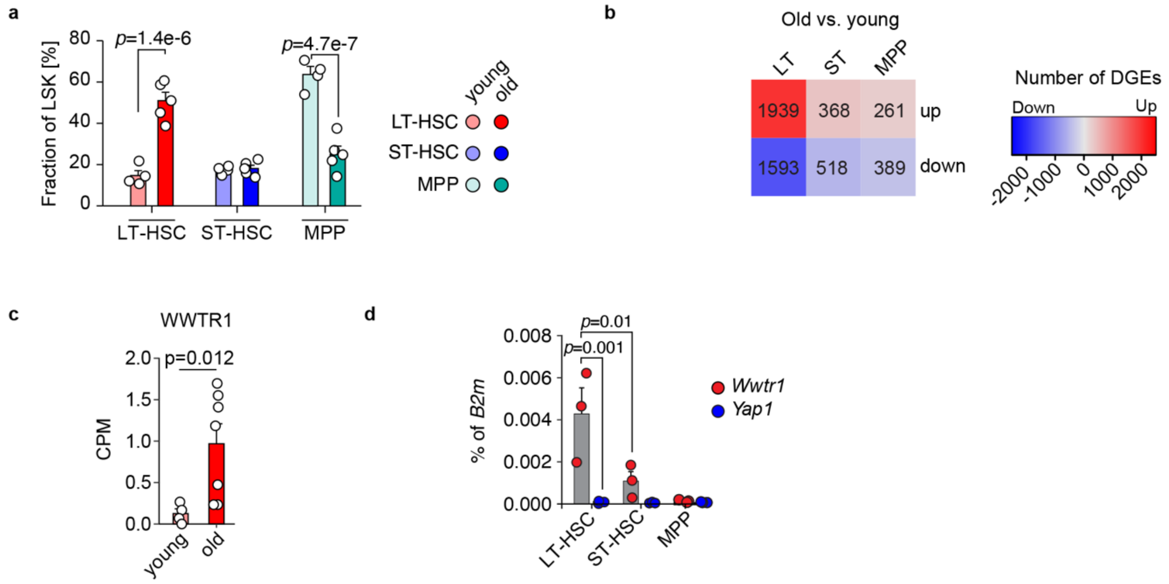

Supplementary Fig. 2: Taz is up-regulated in aging HSCs in mouse and human.

a. Bargraph of flow cytometric analysis of young and old mice. (n=4 for young and n=5 for old, one-way ANOVA with Tukey HSD post hoc test). Data are presented as mean values +/- SEM.

b. Summary of differentially expressed genes comparing old vs. young LT-HSCs.

c. Gene expression of *WWTR1* in HSPCs of young (20-30 years, n=4) and old (50-60 years, n=7) humans. The RNA-Seq count matrix was downloaded from GEO (GSE115348). Welch T-test. Data are presented as mean values +/- SEM.

d. qRT-PCR analysis for *Yap1* and *Wwtr1* expression of old mice. The data were normalized to *B2m* by the $\Delta\Delta$CT Method. (data is from n=3 old animals). One-way ANOVA with Tukey HSD post hoc test. Data are presented as mean values +/- SEM.

Source data are provided as a Source Data file.

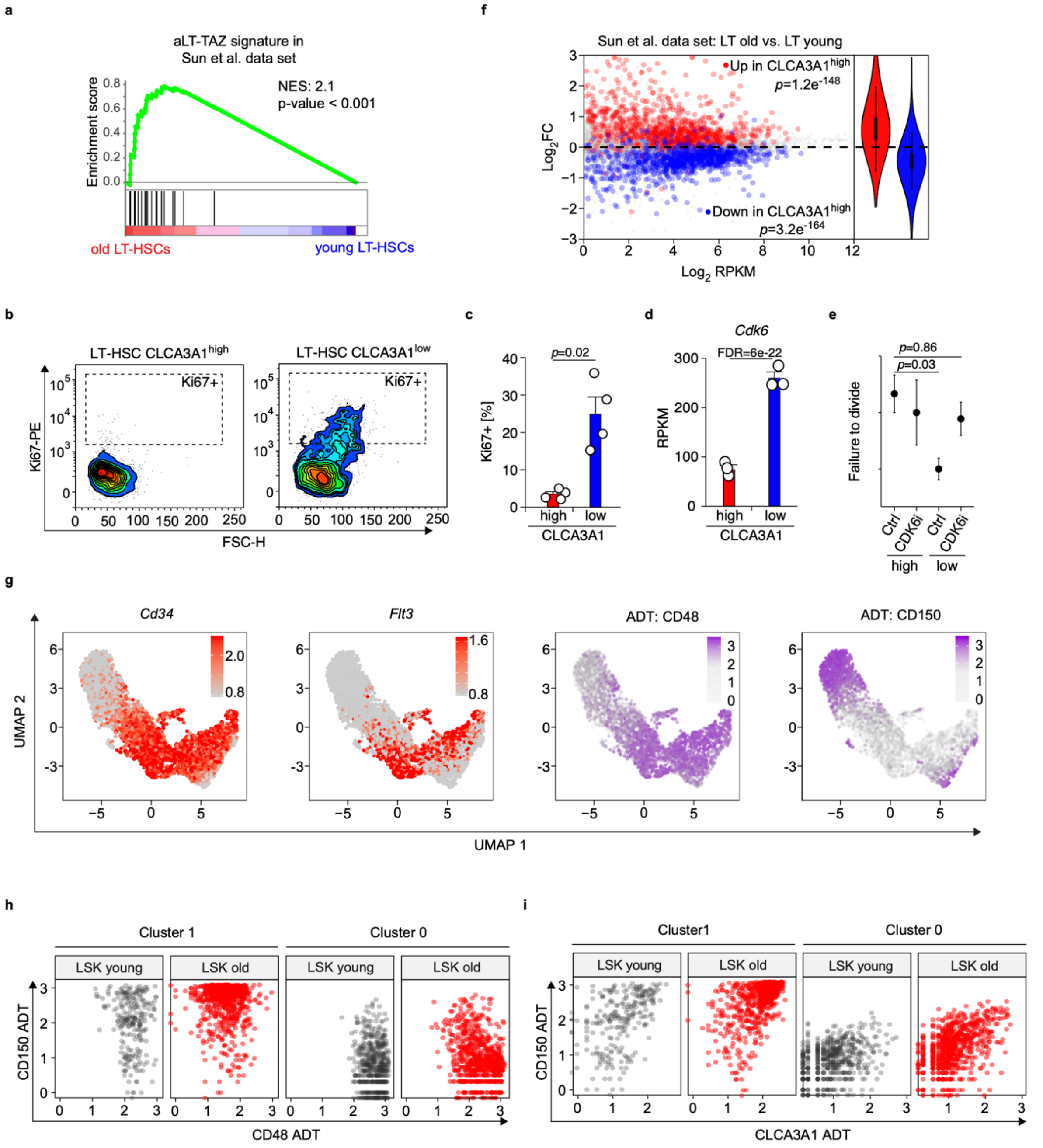

Supplementary Fig. 3: Clca3a1^{low} HSCs resemble young HSCs on the transcriptional level.

a. GSEA analysis of an RNA-Seq study (GSE47817) comparing old vs. young HSCs using the aLT-TAZ signature. NES=normalized enrichment score.

b. Representative intracellular Ki67 flow cytometry plots in Clca3a1^{low} and Clca3a1^{high} CD34^{neg} CD135^{neg} LSK cells.

c. Quantification of samples analysed in b (n=4, one-way ANOVA with Tukey HSD post hoc test). Data are presented as mean values +/- SEM.

d. *Cdk6* mRNA expression from the Clca3a1^{high} vs. Clca3a1^{low} bulk RNA-Seq data set (n=3 per group). RPKM=reads per kilobase per million mapped reads. FDR=false discovery rate. Data are presented as mean values +/- SEM.

e. Cells that failed to enter cell cycle upon plating *in vitro*. Single cells were plated into single wells and cultured for 72 h either with solvent control (Ctrl) or 50 nM of the specific Cdk4/6 inhibitor PD033299 (CDK6i). Cells that failed to divide were counted. N=3 biological replicates. Approximately one hundred cells per condition were counted. (Significance test for negative binomial distribution). Data are presented as mean values +/- SEM.

f. MA plot of differentially expressed genes between old vs. young HSCs. The data was downloaded from GEO (GSE47817). The genes that were up-regulated in $Clca3a1^{high}$ vs. $Clca3a1^{low}$ HSCs are highlighted in red, the down-regulated genes are highlighted in blue.

g. UMAP plots showing the expression of *Cd34* and *Flt3* mRNA (red), and CD48 and CD150 protein level (purple) by antibody-derived tags (ADTs)

h.-i. Signal of the indicated antibody-derived tags (log-transformed data) stratified according to cluster 1 and cluster 0, respectively. Cluster 1 contains LT-HSCs.
Source data are provided as a Source Data file.

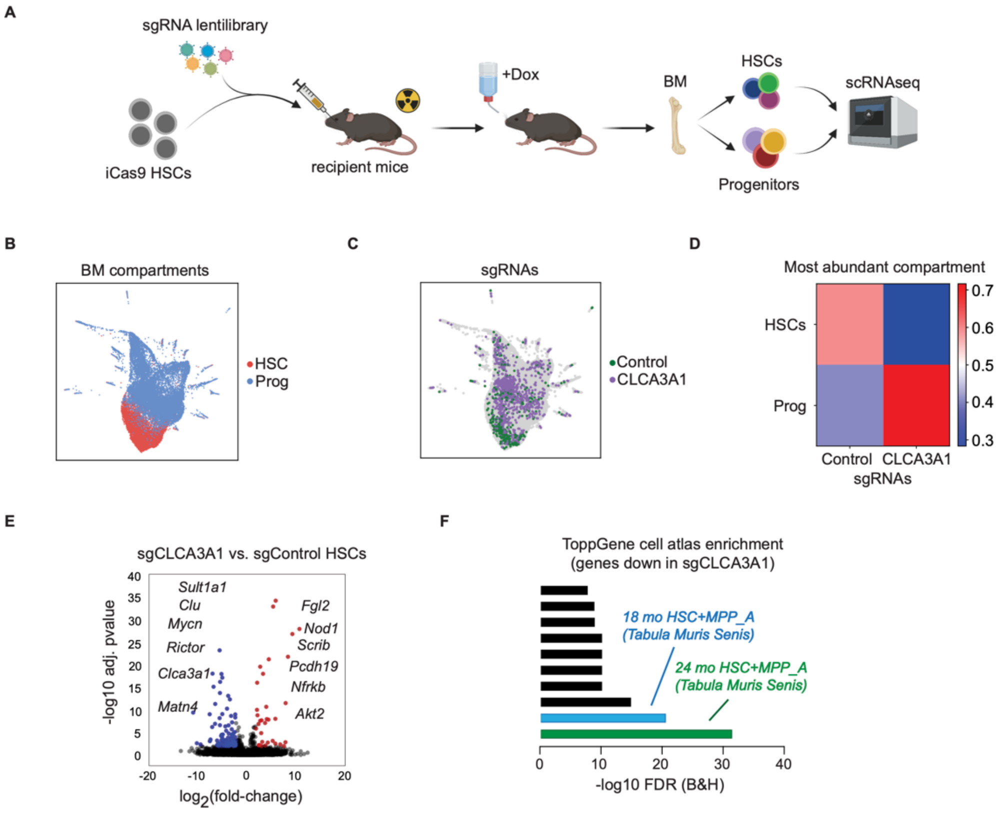

Supplementary Fig. 4: Clca3a1 depletion correlates with increased HSC differentiation and a decreased aged-HSC signatures.

a. Scheme of the *in vivo* CRISPR screening for inhibitors of HSC differentiation. HSCs were purified from 8-10 week-old donor mice and transduced with a CROP-Seq lentiviral library to express sgRNAs and mNeonGreen at an MOI of 0.3. After 8h in culture, transduced donor cells were transplanted in lethally-irradiated recipients. Dox was added in drinking water to induce Cas9 (and gene KO) after stable myeloid engraftment was achieved. Two months after Dox addition, bone marrow cells were isolated from all bones, and mNeonGreen expressing HSCs (CD150^{pos} LSKs) and Progenitors (remainder of c-Kit^{pos} cells) were analyzed by bulk DNA-Seq and scRNA-Seq to measure the distribution of sgRNAs across different hematopoietic compartments.

b. Graph embedding of isolated HSC and Progenitor transcriptomes labeled according to their sample source hematopoietic compartment: HSCs, hematopoietic stem cells. Prog, Progenitors.

c. Graph embedding of isolated HSC and Progenitor transcriptomes labeled according to their corresponding sgRNA sequence (Control and Clca3a1 sgRNA cells are colored green and purple, respectively).

d. Heatmap showing the relative frequency of Control sgRNA cells and Clca3a1 sgRNA cells across each BM compartment. In contrast to control cells, Clca3a1 cells are principally detected in the Progenitor compartment.

e. Volcano plot of the differential expression analysis comparing sgClca3a1 and sgControl HSCs. The plot shows the $\log_2$ fold-change (x-axis) and the negative $\log_{10}$ adjusted p-value (y-axis) for each gene in the analysis. Genes significantly enriched in sgClca3a1 HSCs are highlighted in red. Genes significantly downregulated in sgClca3a1 HSCs are highlighted in blue (*Sult1a1, Clu, Mycn, Rictor, Clca3a1, Matn4*).

f. Enriched signatures downregulated in sgClca3a1 (from the ToppGene cell atlas database). The top two enriched signatures are highlighted and labeled (transcriptome profiling of 18-month-old and 24-month-old LT-HSCs from the Tabula Muris Senis project).

Source data are provided as a Source Data file.

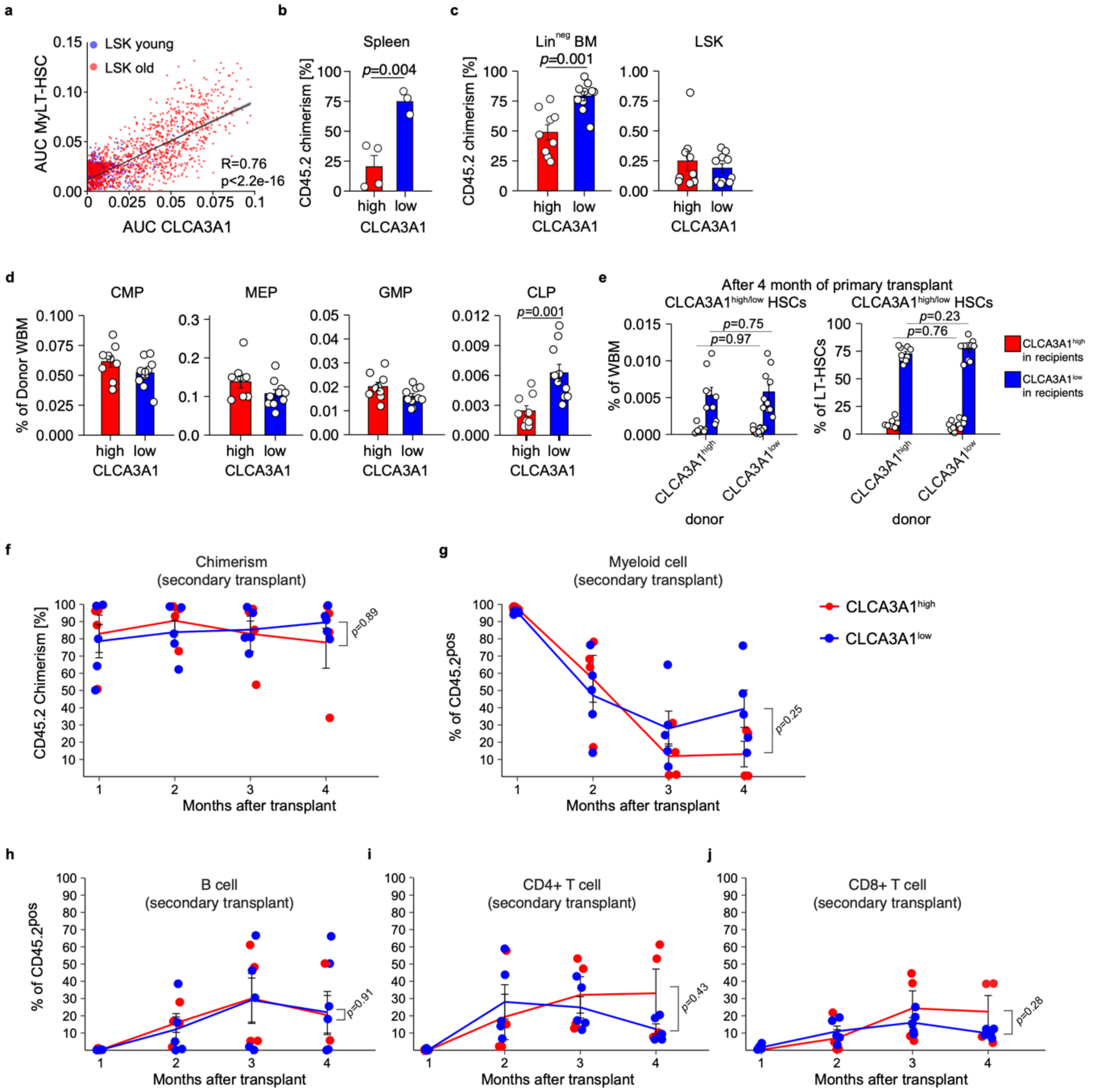

Supplementary Fig. 5: Clca3a1^{high} HSCs show aging phenotypes in primary transplants which get lost in secondary transplants.

a. Scatter plots of Clca3a1^{high} gene set activity (AUC scores) plotted against the MyLT-HSC gene set activity (R=pearson correlation coefficient, linear regression t-test).

b. Leukocyte chimerism in the spleen four months after transplantation (n=3 for Clca3a1^{low}, n=4 for Clca3a1^{high}, one independent transplantation round, two-tailed Welch t-test).

c. Chimerism in the BM in the lineage-negative (Lin^{neg}) or LSK population four months after transplantation (n=10 for Clca3a1^{low}, n=9 for Clca3a1^{high}, two independent transplantation rounds, two-tailed Welch t-test).

d. Percentage of progenitor populations of whole BM (WBM) within the donor compartment (CD45.2^{pos}) four months after transplantation (n=10 for Clca3a1^{low}, n=9 for Clca3a1^{high}, two independent transplantation rounds, two-tailed Welch t-test). CMP=common myeloid progenitor, MEP=megakaryocyte-erythroid progenitor, GMP=granulocyte-macrophage progenitor, CLP=common lymphoid progenitor.

e. Clca3a1^{high} frequency on donor-derived LT-HSCs isolated from recipients which were transplanted with Clca3a1^{high} and Clca3a1^{low} LT-HSCs, respectively. The analysis was performed four months post-transplantation ((n=10 for Clca3a1^{low}, n=9 for Clca3a1^{high}, two independent transplantation rounds, two-tailed Welch t-test).

f. Secondary transplants from bone marrow cells. Leukocyte chimerism (based on CD45.2 expression) in the peripheral blood over time (n=5 for $Clca3a1^{low}$, n=4 for $Clca3a1^{high}$, two independent transplantation rounds, two-way ANOVA with Tukey HSD post hoc test).

g.-j. Percentage of myeloid cells ($CD11b^{pos}$), B cells ($B220^{pos}$) and T cells ($CD4^{pos}$ or $CD8^{pos}$, respectively) within the donor compartment ($CD45.2^{pos}$) over time after secondary transplantation (n=5 for $Clca3a1^{low}$, n=4 for $Clca3a1^{high}$, two independent transplantation rounds, two-way ANOVA with Tukey HSD post hoc t test).

(b.-j.) Data are presented as mean values +/- SEM.
Source data are provided as a Source Data file.

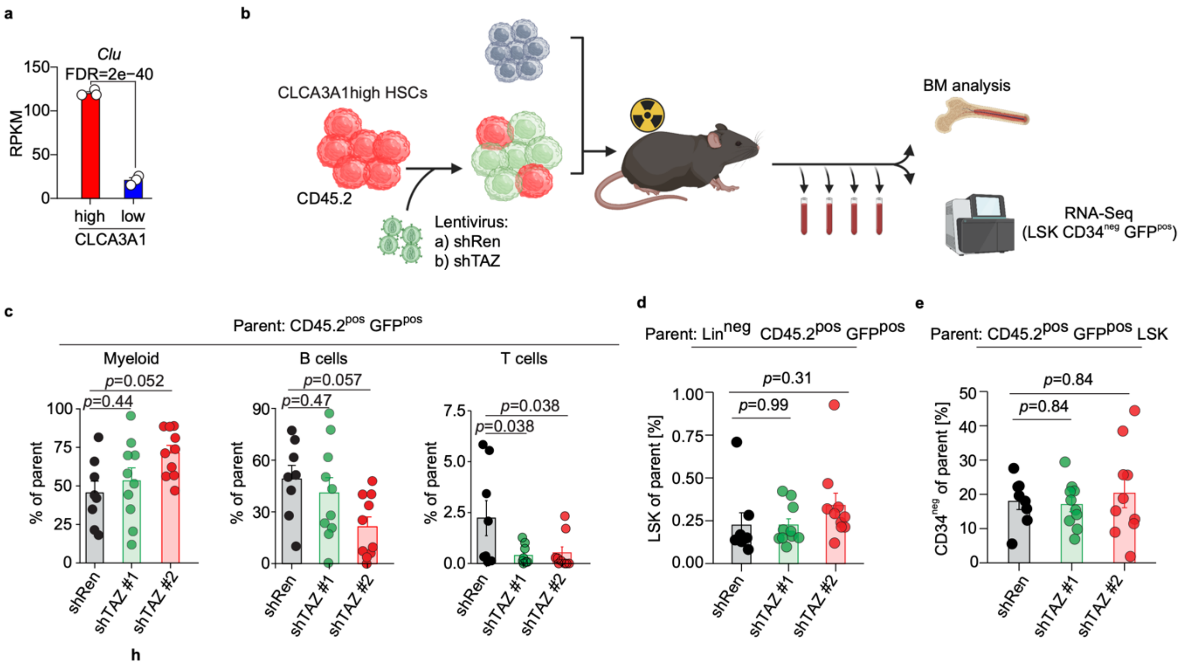

Supplementary Fig. 6: TAZ depletion leads to mild changes myeloid vs. lymphoid output of Clca3a1^{high} HSCs

a. Expression of *Clu* in RNA-Seq data of Clca3a1^{high} and Clca3a1^{low} LT-HSCs, respectively. (n=3 per group).

b. Schematic that describes the experimental approach to deplete Taz in old Clca3a1^{high} LT-HSCs. The infection efficiency was determined from cells kept in culture for three days and set to 100% for zero months after transplantation.

c. PB analysis four months after transplantation of Clca3a1^{high} HSCs transduced with the indicated shRNAs. The cells were gated on CD45.2 GFP^{pos} cells and the distribution of the cells is given as percentage of parent cells (shRen: n=8, shTAZ#1: n=10, shTAZ#2: n=10, three independent transplantation rounds, one-way ANOVA with post hoc paired Wilcox-test and Benjamini-Hochberg correction). Data are presented as mean values +/- SEM.

d.-e. Bar graphs for the percentage of LSK cells (left panel) or CD34^{neg} LSK cells (right panel) in BM within the indicated parent gate (shRen: n=8, shTAZ#1: n=10, shTAZ#2: n=10, three independent rounds of transplantation, one-way ANOVA with post hoc paired Wilcox test and Benjamini-Hochberg correction). Data are presented as mean values +/- SEM.

Source data are provided as a Source Data file.

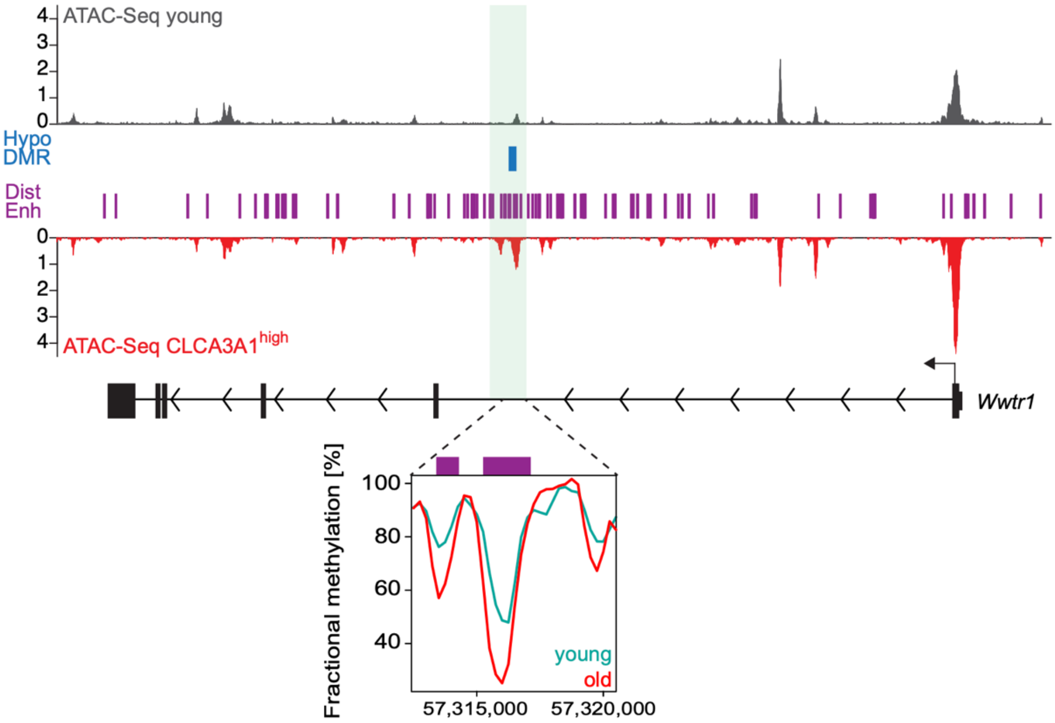

Supplementary Fig. 7: The Taz (*Wwtr1*) locus becomes hypomethylated during aging

Schematic of the *Wwtr1* locus. Regions demonstrating significant DNA hypomethylation (Hypo DMR) in aging HSCs (GSE47815) are shown in blue. Candidate enhancer regions (Dist Enh; taken from ENCODE) are shown in purple. ATAC-Seq tracks for young (grey) and Clca3a1^{high} (red) LT-HSCs are plotted. A blow-up of the significantly hypomethylated enhancer region, which also demonstrates increased chromatin accessibility in Clca3a1^{high} HSCs, and the corresponding fractional DNA methylation is shown below. Source data are provided as a Source Data file.

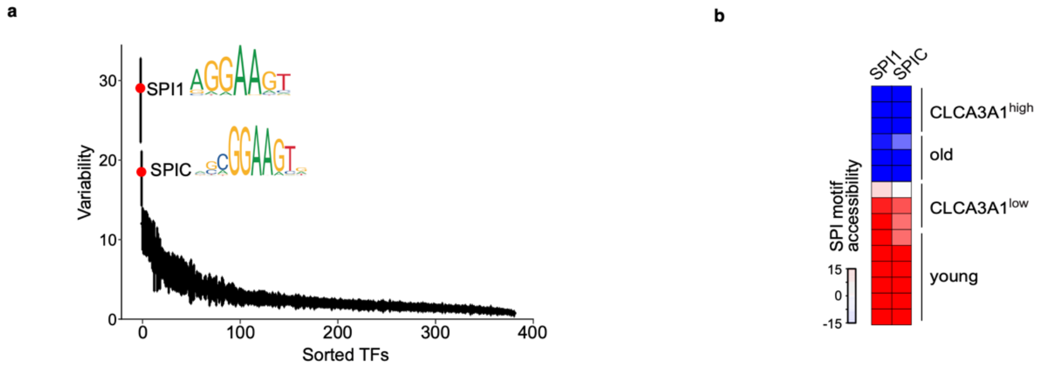

Supplementary Fig. 8: Chromatin accessibility of SPI1 decreases during HSC aging

a. Motif analysis of the most variable motifs in FAST-ATAC peaks of Clca3a1^{high} vs. Clca3a1^{low} LT-HSCs, respectively. The top two motifs are highlighted, which all contain variations of the PU-box motif 5'-GAGGAA-3'.

b. Accessibility of SPI1 and SPIC motifs in Clca3a1^{high}, Clca3a1^{low}, oLT-HSCs and yLT-HSCs.

Source data are provided as a Source Data file.

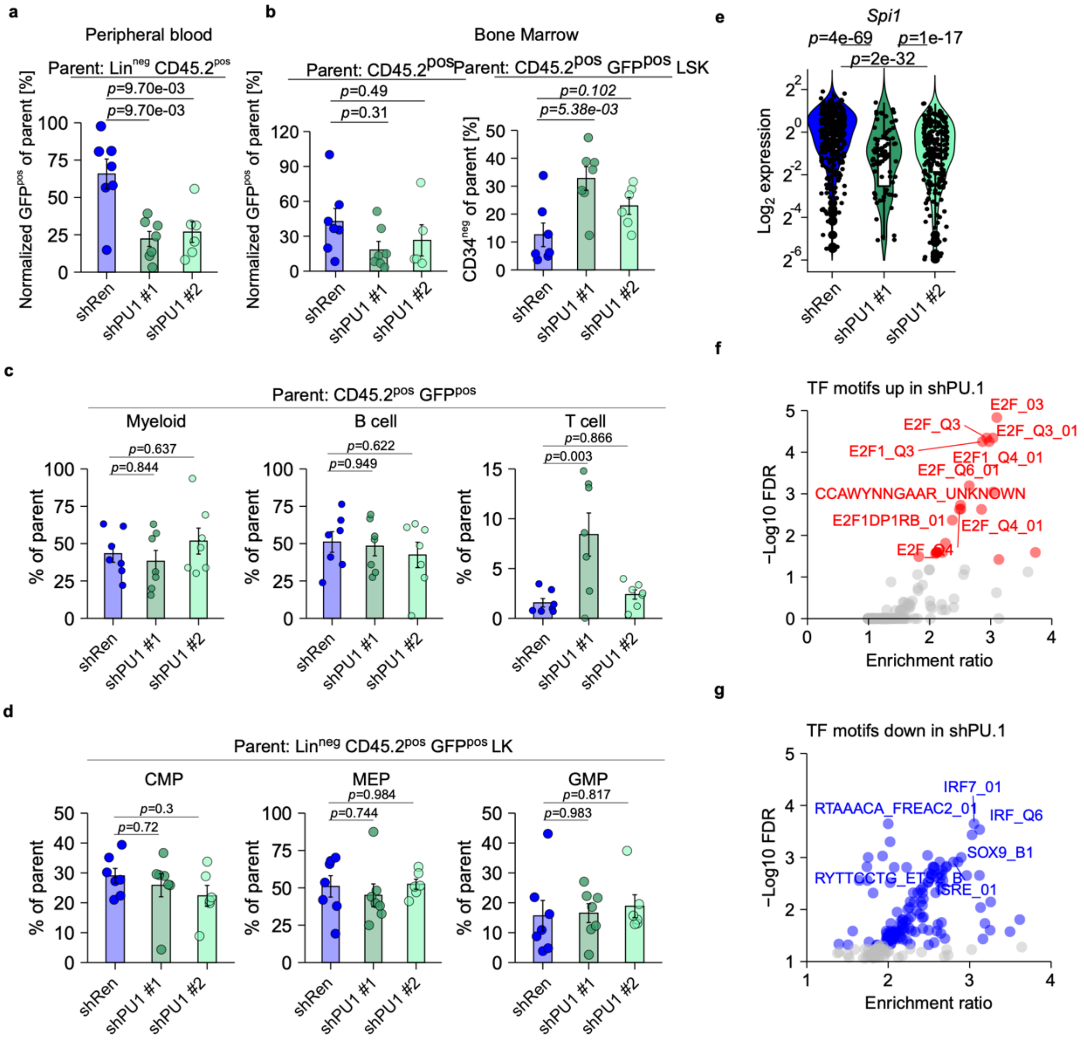

Supplementary Fig. 9: Peripheral blood, bone marrow and motif analysis in LT-HSCs of shPU.1 transplantation

a. PB analysis after transplantation of yHSCs transduced with the indicated shRNAs. The GFP^{pos} population in the donor CD45.2^{pos} compartment was analyzed two months post-transplantation (shRen: n=7, shPU1#1: n=7, shPU1#2: n=6, two independent transplantation rounds, one-way ANOVA with post hoc paired Wilcox-test and Benjamini-Hochberg correction).

b. BM analysis two months post-transplantation of yHSCs transduced with the indicated shRNAs. The CD34^{neg} LSK population in the donor CD45.2^{pos} GFP^{pos} Lin^{neg} compartment was analyzed as percent of LSK (shRen: n=7, shPU1#1: n=7, shPU1#2: n=6, two independent transplantation rounds, one-way ANOVA with post hoc paired Wilcox-test and Benjamini-Hochberg correction).

c. Peripheral blood analysis two months post-transplantation of Clca3a1^{high} HSCs transduced with the indicated shRNAs. The cells were gated on CD45.2 GFP^{pos} cells and the distribution of the cells is given as percentage of parental cells (shRen: n=7, shPU1 #1: n=7, shPU1 #2: n=7, two independent transplantation rounds, one-way ANOVA with post hoc paired Wilcox-test and Benjamini-Hochberg correction).

d. Bar graphs for the percentage of CMP, MEP and GMP in the Lin^{neg} CD45.2^{pos} GFP^{pos} bone marrow fraction (shRen: n=7, shTAZ#1: n=7, shTAZ#2: n=6, three independent transplantation rounds, one-way ANOVA with post hoc paired Wilcox-test and Benjamini-Hochberg correction).

e. Violin plots of *Spi1* expression in all LSK cells in the different shRNA populations (shRen: n=7, shPU1 #1: n=7, shPU1 #2: n=7, two independent transplantation rounds, one-way ANOVA with post hoc paired Wilcox-test and Benjamini-Hochberg correction). Boxplots: bottom/top of box: 25th/75th percentile; upper whisker: min(max(x), Q_3 + 1.5 * IQR), lower whisker: max(min(x), Q_1 – 1.5 * IQR), centre: median

f-g. Overrepresentation motif analysis using Webgestalt for the genes that were differentially expressed in the shPU.1 HSC scRNA-Seq data set (shPU.1 vs. shRen specifically in LT-HSCs). Source data are provided as a Source Data file.

Supplementary Table 1: Gene sets

Gene sets (gene symbol)													
TAZ-induced	aLT-TAZ	aHSC	myLT-HSC	Clca3a1_up	LT-HSC Kim_et_al	LT-HSC Cabezas-Wallscheid_et_al	ST-HSC	MPP	Conserved LT-HSC	E2F targets	Replication	Recombination	Bund by PU1 and down after shTAZ
Ace	Aldh3a1	Abcb1a	Dnm3	Gm16548	Hbb-bs	Smtnl1	Ly6c2	Wfdc17	Tcf3	Asf1b	Ache	Actl6a	Slk
Chrnb1	Ccbe1	Abcb1b	Ptafr	Fam110c	Gm15915	Arxes1	Gm13031	Gm20871	D10Ertd398e	Aurka	Atm	Actr5	Rprd1a
Aldh3a1	Clca3a1	Acpp	Thsd4	Col16a1	Epdr1	Glis1	Kifc1	Zfp981	Htf9c	Brca1	Atr	Actr8	Haus2
Alox5	Wwtr1	Acsl4	Gstm2	Mylk	Art4	Gfi1	Espl1	Akna	Rbp1	Bub1	Atrx	Apex1	Ssx2ip
Rhod	Amotl2	Adgrg2	Tm4sf1	Cdcp1	Hba-a2	Gm15034	Slco3a1	Hbb-bs	Ocln	Bub1b	Bard1	Apex2	Stom
Bgn	Rnf39	Alcam	Gpx3	Art4	Ly6a	Sema6d	Nusap1	Ano10	C80638	Cbx2	Baz1a	Atm	Dcp2
Bmp2	Ret	Amotl2	Sbspon	Prn	Aldh1a1	Inhba	Cdca2	9030619P08Rik	Mpdz	Ccna2	Blm	Atrx	Eea1
Anxa2	Vwf	Arhgef28	Gpr183	Abca4	Aplp2	Atf7ip2	Fam64a	B3gnt5	Gprc5b	Ccnb1	Brca1	Bard1	Dusp16
Car8	Mfsd7a	Art4	Gprc5c	Selenom	Aqp1	Shd	Alas1	Prr5	Sparcl1	Ccne1	Brca2	Batf	Fam193b
Car2	Lpl	B3galt1	Cadm1	Ak1	Atp1b2	Slamf8	Ap3s1	Hba-a2	Smpd1	Ccnf	Brip1	Bcl11b	Me2
Serpinh1	Unc5b	Bmpr1a	Ramp2	Alcam	Car1	Dnm3	Birc5	Cd53	Procr	Cdc20	Ccne1	Blm	Cep295
Cd24a	Meis3	Casp12	Dhrs3	Ampd3	Cdkn1c	Neo1	Bub1b	Cd69	1110007A14Rik	Cdc6	Ccne2	Brca1	Mmab
Cd9	Krt7	Cavin2	Selp	Apoe	Cish	Gm9378	Runx3	Ctss	Pclo	Cdc7	Cdc25a	Brca2	Il6ra
Cdkn1c	Rhod	Cd200r4	Gda	Bmp4	Cpox	Cyp26b1	Ccnb2	Emb	Ptpn21	Cdca3	Cdc25c	Brip1	Zfp526
Clca3a1	Gem	Cd38	Ehd3	Cd38	Epor	A3galt2	Ccnf	P2ry14	Mm.35515	Cdca5	Cdc34	Cdc7	Cog3
Cnn2	Rab40b	Cd63	Clca3a1	Chrm3	Mecom	Gimap3	Cd34	Flt3	Agpt	Cenpf	Cdc6	Cntd1	Slc30a9
Col4a5	Serpinh1	Cd9	Ptprk	Clca3a1	Fhl1	Pianp	Cd48	Gem	2210402G22Rik	Cenph	Cdc7	Dclre1c	Ccdc130
Csrp3	Slfn2	Chrna7	Vwf	Cldn5	Gda	Zfp711	Cd53	H2-Ob	Efnb2	Cenpk	Cdk2	Dmc1	C130036L24
Cyb561	Rin1	Clca3a1	Gm9199	Clu	Grb10	Loxl2	Cdk6	Hbb-b2	AI837850	Cenpq	Cdk2ap1	Eme1	Rik
Dr1	Arhgef17	Cldn12	Nupr1	Efna1	H2-Aa	Sel1l3	Cdkn2c	Hbb-b2	Jcam2	Chaf1b	Cdk9	Eme2	Xrcc2
Efnb2	Efnb2	Clec1a	Clec1a	Eps8	H2-Eb1	Klrb1c	Cebpa	Irf8	Elavl4	Dck	Cdt1	Endog	Gadd45a
Fcer1a	Cdkn1c	Clec1b	Med1	Ezh1	Hbb-b2	Clec7a	Cpa3	Il12a	Rras	Depdc1a	Chaf1a	Ercc1	Malt1
Fgf3	Hoxa5	Clu	Jam2	Fgfr3	Hbb-b2	Fut4	Csf2rb	Il18rap	Mm.41339	E2f3	Chaf1b	Ercc4	Zfp217
Gata2	Chrnb1	Cntn1	Rorb	Fhl1	Jchain	Gm11948	Csf3r	Il1r1	AI842353	Ezh2	Chek1	Exo1	Ankrd13d
Gdf10		Cpne8	Gpr64	Gda	Cd74	Efna1	Cst7	Il6ra	Mm.37710	F13a1	Chtf18	Exosc3	Tmem206
Gem		Cxcl16	Piscr2	Ghr	Itsn1	Nipal2	Ctsg	Itgb7	Mm.74203	Fam111a	Cinp	Fancm	Mdm1
Gp1bb		Cyb561	Muc1	Gpx3	Klf1	D230022J07Rik	Ect2	Lsp1	Pphn	Fbxo45	Clspn	Fen1	Slc25a10
Gria4		Cyp26b1	Jun	Gstm2	Lhcgr	Meg3	Emid1	Mpeg1	5730557K01Rik	Fen1	Dbf4	Gins2	Pcx
Gzmb		Cysltr2	Bmp4	Hoxb5	Ltb	Fbxo2	Fgf3	Ncf1	Jcam3	Fignl1	Dnaja3	Gins4	Ccdc134
Gzmc		Cyyr1	Vmp1	Hoxb6	Mt1	Exph5	Foxm1	Ngp	Mm.141984	Gp1ba	Dtl	H2afx	Shmt1
Gzme		Ddr1	Itgb3	Inha	Mt2	Mfsd7a	Fut7	Notch1	AA986099	H2afx	Dut	Hfm1	Lyrm9
Gzmf		Dennd5b	Osmr	Itgb3	Ndn	Tenc1	B4galnt1	Pgr	Ches1	Hmgb2	E4f1	Hmgb2	Odr4
Gzmg		Dhrs3	Myo1e	Klrb1c	Ngp	Ncmap	Cdca3	Dhrs3	P2rx4	Hmmr	Egf	Hus1	Cntrob
Heph		Dnm3	Wwtr1	Tm4sf1	Nkx2-3	Igf2	Hdc	S100a8	Hoxa5	Incenp	Exo1	Ighmbp2	Plxnd1
Hoxa10		Dsg2	Epas1	Matn4	Npdc1	Zfp37	H2afx	S100a9	Fkbp7	Iqgap3	Fam111a	Kin	Zc3h8
Hoxa3		Efna1		Ncam2	Abcb4	Sox9	Hmmr	Satb1	Mtap7	Kif11	Fancm	Klhdc3	Lonrf1
Hoxa5		Ehd3		Ndn	Pklr	Pde6b	Igfbp4	Ccl9	Aldh2	Kif20a	Fen1	Lef1	Mxd1
Igf2r		Enpp5		Neo1	Procr	Setbp1	Itgal	St8sia4	Nbea	Kif2c	Gins1	Lig1	Ddx59
Cyr61		Evc		Mycn	Cyth3	Gm13594	Kif17	Snn	Slc12a2	Lmnb1	Gins2	Lig3	Tsacc
Igfbp5		Exoc6b		Npdc1	Pygm	Gbp9	Kif4	Stxbp4	AI788588	Mapk8	Gins3	Lig4	Srl
Kcna4		Fap		Pbx1	Pygm	Slc17a8	Lgals1	Tbxa2r	Mm.33054	Mcm3	Gins4	Mcm8	Syvn1
Kcnk2		Fhl1		Pde9a	S100a8	Gm23706	Ms4a3	Dntt	4931406C07Rik	Melk	Helb	Mcrs1	Tent4a
Lmna		Gabra4		Plscr2	S100a9	Syt13	Mc5r	Thbs1	AI836256	Mki67	Hmga1	Mlh1	Gm2011
Lpl		Gem		Prnp	Slfn2	Igf1	Melk	5031439G07Rik	Rps4x	Myb	Hus1	Mlh3	Crebrf
Mcpt1		Ghr		Pros1	Sphk1	Nova1	Mpo	Gpr171	Lama5	Nasp	Igf1	Mnd1	E2f7
Mcpt2		Gm10419		Pygm	Sult1a1	Npas2	Nek2	BC035044	Rpl7	Ncapd2	Ighmbp2	Morf4l1	Serac1
Mcpt8		Gpr183		Pygm	Mgst2	Zfr2	Palm	Cd52	Vdr	Ncapg	Ing4	Msh2	C130046K22
Mif		Gstm2		Bex1	Tbxas1	Ccdc136	Pdgfrb	Lax1	1200003C15Rik	Ncapg2	Ing5	Msh3	Rik
Meis3		Hpgds		Rorc	Trim47	Ocln	Pgam1	Fgd2	Evi1	Ncaph	Kctd13	Msh5	Man1c1
Myom1		Id2		Ryk	Trib2	Ptges	Prg2	H2afy	Slc2a8	Nr1d1	Kin	Msh6	Caprin2
Myt1		Il1rapl2		Cavin2	Tgm2	Gm13111	Prtn3	Tpm4	Matn2	Nup50	Lig1	Mus81	Eri2
Nap1l2		Itgb3		Selp	Thbs1	Wnk4	Ccl9	Bex6	Ptprr	1700123L14Rik	Lig3	Nbn	Tjp1
Mycn		Jam2		Plk2	Tnfaip2	Gm6939	Sell		Col18a1	Nusap1	Lig4	Ncoa6	Iah1
Ntn1		Jun		Sult1a1	Tfrc	Gm13156	Aurkb	Gm5111	Cyp7b1	Pbk	Mcm10	Nfrkb	Rbbp8
Pde6b		Kcnip3		Bhlhe40	Upp1	Errfi1	Aurka	Slc9a9	Mapk13	Plk1	Mcm2	Nono	Gramd4
Pdgfa		Kdr		Tns2	Vwf	Disc1	Kif15	Milr1	Vps35	Prc1	Mcm3	Nsmce1	Kctd6
Pou2f3		Klrb1b		Cracr2b	Slc27a6	Plekhg1	Kcnk12	Shisa8	Chst1	Prim1	Mcm3ap	Nsmce2	Msh5
Ptprf		Klrb1c		Arhgap29	Tspan33	Sdc4	Hk3	Parp8	Hn1	Psip1	Mcm4	Palb2	Vps8

Ret		Lamp2		Slc17a8	Blvrb	Pbld1	Fam46a	Rasa4	Ereg	Racgap1	Mcm5	Parp1	Rnf19b
Rpl32		Ldhd		Hid1	Gimap8	Hoxa3	Fam117a	Tspan6	Hsd11b2	Rad51ap1	Mcm6	Paxip1	Stx2
Rpl32		Lpar6		Trim47	Adgre5	Unc45b	Depdc1b	Prr33	Siat8d	Rad54l	Mcm7	Pcna	Slc45a4
Rpl9		Lpl		Cdc42bpb	Ermap	Gbp6	Dlgap5	Cep85	Mta1l1	Rbbp8	Mcm8	Pola1	Uimc1
Rps17		Lrrn1		Tgm2	Asns	Maf	Arl11	Scpep1os	Scya27	Rbl1	Nasp	Pola2	
Rps19		Lsr		Phactr1	Abcg3	Sfrp1	Top2a	Scpep1	Ltbp1	Rpl3	Nbn	Polb	
Scin		Ly6e		Phf11d	Samd12	Spin4	Trf	Jakmip1	Fmn2	Shcbp1	Ncoa6	Pold1	
Scn5a		Mmp14		Vdr	Bex4	Chi3l7	Ttk		Large	Skp2	Nfia	Pold2	
Slfn2		Muc1		Vwf	Snhg14	Amigo2	Tyrobp		Rps3	Smc2	Nfic	Pold3	
Snca		Muc13		Slc27a6	Iigp1	Cd74	Arhgap11a		Tuba3	Syce2	Nfix	Pold4	
Serpine2		Myo1e		Myof	Gbp10	Ifitm7	Arhgef39		Lactb	Tacc3	Nol8	Pole	
Src		Nckap1		Sbspon	Hacd4	1810062G17Rik	Sh2d5		Ahsg	Tk1	Nt5m	Pole2	
Timp3		Ndrg1		Acer2	Pdzk1ip1	Slmo1	Plac8		Parva	Top2a	Nup98	Polm	
Vwf		Neo1		Fgd5	Fam132a	Actr3b	BC035044		Eif3s7	Tpx2	Parp1	Poln	
Wnt10b		Npdc1		Clec9a	Plxdc2	Gm19684	Ffar2		Gla	Tyms	Parp2	Polq	
Plagl1		Nrg4		Crebl2	Zfp467	F830016B08Rik	Prc1		Wdr10	Ube2c	Parp3	Prim1	
Asns		Ntf3		Klhl4	Mllt3	Slc25a47	H2afy		Ak1	Ube2t	Pcna	Prkdc	
Syn3		Ocln		Gas2l3	Prdm16	Atp9a	Slc28a2		Acadvl	Usp1	Pdgfb	Psmc3ip	
Slc14a2		Osmr		Irf2bpl	Mmrn1	Nr3c2	Flnb		Pln		Pnkp	Psmd14	
Rragd		Pdgfd		Gadd45g	Mamdc2	Procr	Bin1		AI854032		Pola1	Rad21	
Adgre4		Perp		D630039A03Rik	Ces2g	Mx1	Hist1h2ac		D15Wsu77e		Pola2	Rad50	
Tuba8		Pgr		Clec1a	Tbkbp1	Runx1t1	Hist1h2ae		Zfp354a		Polb	Rad51ap1	
Rps28		Pla2g4a		Mtss1l	Slfn5os	Plekhg6	Hist1h2ag		0610039D01Rik		Pold1	Rad51c	
Gp9		Plcl1		Ccdc60	Obsl1	Trib2	Hist1h2ah		Laf4l		Pold2	Rad52	
Amotl2		Plek		Slamf1		Srpk3	Hist1h2ab		Six5		Pold3	Rad54b	
Cdc42ep4		Plscr1		Rasgef1b		Gm11100	Hist1h2af		Hp1bp3		Pold4	Rad54l	
Pf4		Plscr2		C530008M17Rik		Chst2	Hist1h2ai		Cntn1		Pole	Rag1	
Rps27		Plscr4		Gpr183		Cpne8	Gm16897		Jag2		Pole2	Rag2	
Tesc		Ppp1r16b		Bex4		Pde1b	Bex6		Myom1		Pole3	Rbbp8	
Rpl23		Prcp		9330175M20Rik		Sult1a1	Ubash3a		Mm.826		Polg	Rbm14	
Ndufa3		Prnp		Nckap1		Gm15880	Elane		Nr0b1		Polg2	Recql	
Chchd1		Procr		Lsr		Usp27x	Slc7a8		Aqp4		Polh	Recql4	
Cox7b		Prtn3		Irf6		Tead3	Knstrn		Eya2		Poli	Recql5	
Rps21		Ptger4		Ctnnal1		Cd28	Pbk		Mm.33003		Polk	Rfc1	
Rpl36al		Ptprk		Ramp2		Gkn3	Cdca8		Epha5		Poll	Rfc2	
Rpl35		Rab34		Gm5833		Neil2	Adgrg3		5730454C12Rik		Poln	Rfc3	
Cap2		Ramp2		Tbc1d8		Dok3	Plek		Krt2-18		Polq	Rfc4	
Snap47		Rdh10		Nupr1		Gm10499	Trip4		4930443C24Rik		Prim1	Rfc5	
Rps27l		Rgn		Amotl2		Tmem136	Lat2		Mm.180366		Pura	Rmi1	
Rpl24		Rhoj		Bcam		Dync1i1	Sdf2l1		Mm.178672		Rad1	Rnf138	
Diras2		S100a6		Ehd3		Kazn	Serpinb1a		Atp1b1		Rad17	Rnf168	
Tmbim4		Selp		Sqor		Gm4951	Spc25		Tie1		Rad50	Rnf8	
Mrpl14		Sema7a		Il1rapl2		Clip3	Nuf2		Ptprz		Rad9a	Rpa1	
Mtcl1		Sfrp1		Fads3		Glipr1l1	Ndc80		Pcp4		Rad9b	Rpa2	
Klk10		Slamf1		Cldn12		Slc16a9	Tespa1		1700023A18Rik		Rbbp6	Rpa3	
Megf10		Slc14a1		Hacd4		Lnx1	Cdca5		Adarb1		Rbbp7	Rpain	
Atoh8		Slc6a15		Tmem246		Neb	2810417H13Rik		Psmb4		Rbbp8	Rtel1	
2010300C02Rik		Stom		Jam2		Sox5	Ube2c		1700019E19Rik		Rbm14	Ruvbl1	
Oraov1		Stxbp4		Evc2		Rhbdf1	Tnfaip8l2		Xrcc1		Rbms1	Ruvbl2	
1810041L15Rik		Tacstd2		Smim6		Pde6h	Kif18b		Bmp8a		Recql4	Setx	
Hoxaas3		Tgm2		Zg16		Efcab4a	Ckap2l		4921537D05Rik		Recql5	Sfpq	
Bmper		Thbd		Cysltr2		Mmp28	Tpx2		Hegfl		Repin1	Smc5	
Ccsap		Tm4sf1		Mllt3		Mapre3	Nkg7		Mad5		Rev3l	Smc6	
Gadl1		Tmem176a		Mmrn1		Zfp612	Kif2c		D8Bwg1112e		Rfc1	Spo11	
Irak3		Tmem215		Pdgfd		Dnajb5	Mdga1		Crim1		Rfc2	Supv3l1	
Nmnat3		Tmem47		Rbpms2		Pygm	Spns3		Prkcq		Rfc3	Swap70	
Chpf		Tnfsf10		Tmem158		Myof			9330160G10Rik		Rfc4	Sycp1	
Rps25		Trpc1		Rin2		Tinagl1			5730555F13Rik		Rfc5	Tcf3	
Vmn2r-ps54		Trpc6		Ifitm7		Mgst2			D4Wsu24e		Rmi1	Tcf7	
Pcolce2		Vldlr		Spaca6		Ryr3			5730406O18Rik		Rnaseh2a	Tep1	
Csgalnact2		Vmp1		Calml4		Traf1			AW108467		Rpa1	Tex11	
Mctp1		Zg16		Exoc6b		3222401L13Rik			Fzd4		Rpa2	Tfpt	
Hemgn		Bcl6		Ift43		Sgsm1			Sprr2a		Rpa3	Top2a	
Wwtr1		C4b		Clca3a2		Clec1a			Sdccag28		Rpain	Top2b	
Spon2		Casp12		Jam3		Mks1			Nnp1		Rrm1	Top3a	

Airn		Cd200r4		Enpp5		Osgin1			Ndr2		Rrm2	Trex1	
Rcor2		Chrna7		Tinagl1		Magi2			Krt1-c29		Rrm2b	Trip13	
Tecpr2		Cyp26b1		Wwtr1		Aim1			Gdap1		Rtel1	Tsn	
Fam84a		Lpl		Rdh10		Card10			2900070I05Rik		Sin3a	Ube2b	
Unc5b		Nupr1		Obsl1		3110001I22Rik			Tapbp		Sirt1	Uchl5	
Snrpd2		Pla2g4a				Wfdc2			Blr1		Slbp	Ung	
Adamts6		Ptger4				Bcam			Igf1r		Slc25a33	Wdr48	
Lmo1		Selp				Gas6			Cldn3		Smarcal1	Wrn	
Krt7		Sema7a				Mboat2					Ssbp1	Xrcc1	
Sp7		Slamf1				Upp1					Ssrp1	Xrcc2	
Mvd		Tgm2				Nkx2-3					Supt16	Xrcc3	
Reps2		Zfp36				Cystm1					Tbrg1	Xrcc4	
Arhgef17		Abcb1a				Bcl6b					Tfam	Xrcc5	
Col28a1		Btg2				Gbp8					Thoc1	Xrcc6	
Wnt9a		Fap				Ikzf4					Timeless	Yy1	
Wrap53		Fhl1				Hoxb5					Tipin	Zfyve26	
Rab40b		Gadd45g				Cx3cl1					Tnfaip1		
BC022687		Gem				Prex2					Top1		
Slc26a8		Id2				Gm13822					Top1mt		
Rin1		Jun				Csf2rb2					Top2a		
Ablim2		Nupr1				Sh3d21					Top2b		
Ces2e		Ptprk				Gm16565					Top3a		
Pde11a		Sfrp1				Tesk1					Topbp1		
Mfsd7a		Stxbp4				Trib3					Trex1		
Rps15a		Bcl6				Zfyve9					Upf1		
Rpl23a		Egr1				Il4					Wrn		
Ltbp1		Mllt3				Evc					Wrnip1		
Rps19-ps3		Pla2g4a				H2-Eb1					Zranb3		
Rpl17		Abat				1600029O15Rik							
Msrb3		Aldh1a1				Chd7							
Ccbe1		Ampd3				Sox18							
Tpm4		Arhgap29				Pabpc4l							
Lgr6		Asb4				Jhdm1d							
Mafa		Aspa				P2rx7							
Pdp1		Cytip				Gm10651							
Rnf39		Dhx40				Rassf6							
Fam84b		Fhdc1				Slc9a3r2							
Ndufs5		Fyb				Gprc5a							
Gm7694		Gda				Vsig2							
Gm7694		Gipc2				Gm5148							
Gm7694		Gpx3				Pou2f2							
Gm7694		Gstm1				Lurap1							
Gm7694		Gstm7				Mmp16							
Gm7694		Klhl4				Mx2							
Gm11651		Mab21l2				Gm12407							
Bbip1		Maf				Hivep3							
		Matn4				Gm12258							
		Mef2c				Abcg3							
		Meis2				2410080I02Rik							
		Mmrn1				Eps8l2							
		Mt1				Mllt3							
		Ndn				Irf6							
		Nt5c3				Tcp11l2							
		Oxr1				Xkr6							
		Pbx3				Rab20							
		Pcdhb16				Vegfc							
		Pclo				Spo11							
		Phactr1				Rasip1							
		Phf11d				Sbf2							
		Pros1				Mir1192							
		Rbpjl				Zfp467							
		Rorb				Rilp							
		Rorc				Tlr2							
		Runx1t1				Fam131a							
		Sbspon				Sh3yl1							

		Sdpr<br>Serpinb6a<br>Serpinb8<br>Sult1a1<br>Tbc1d8<br>Tc2n<br>Tdrd9<br>Tmem56<br>Tox<br>Trim47<br>Tsc22d1<br>Vwf<br>Wwtr1<br>Zfp334<br>Zswim5				Egln3<br>Inha<br>Art4<br>Mgst1<br>Ptgs2<br>Btc<br>Hid1<br>2010016I18Rik<br>Pde9a<br>Camk2b<br>Tnip3<br>Slfn2<br>Rin2<br>Rusc2<br>Airn<br>Glis2<br>Mir3068<br>Vps25<br>Coro2b<br>Insrr<br>Ppp1r16b<br>Chst15<br>Hdac11<br>Agpat4<br>Neu1<br>Elmo3<br>Nbea<br>Pvrl4<br>Erp27<br>Wdr78<br>Ctsf<br>Gm7676<br>Gm973<br>Ccdc60<br>Reps2<br>Irf8<br>Satb1<br>H2-K2<br>5830444B04Rik<br>Igf2bp2<br>Ifitm1<br>Gm11696<br>Igf2r<br>Sqrdl<br>Gm19967<br>Tmem38a<br>Samd12<br>Tox<br>R74862<br><br>S100a4<br>Pim3<br>Arhgap28<br>Zfp583<br>Prnp<br>Gimap5<br>Gm12892<br>Arl10<br>Zbtb4<br>Tspyl2<br>Ghr<br>Rhbdf2<br>Snn<br>Agap2<br>Mageh1							

						Dnhd1<br>Kptn<br>Gm20467<br>Sytl1<br>Ehd2<br>Mecom<br>Prkcb<br>Fmo5<br>2900052L18Rik<br>Tiparp<br>Zfp764<br>Lhcgr<br>Gm15411<br>Rarb<br>Cd200<br>Gimap8<br>Dock6<br>Nav2<br>Fgd5<br>Gm15651<br>Fam84b<br>Hif3a<br>Slc39a13<br>Schip1<br>Gstm1<br>Mvd<br>Inadl<br>Tnks1bp1<br>Zfp831<br>Adarb1<br>Obsl1<br>Trim14<br>Vdr<br>Gda<br>Fads3<br>Tmcc1<br>E230001N04Rik<br>Rgl1<br>Nabp1<br>Camk2d<br>Crebl2<br>Eepd1<br>Fcgrt<br>Ccdc8<br>Mfap2<br>Neurl3<br>Ephx1<br>Ccdc157<br>Nrk<br>Ecscr<br>D630039A03Rik<br>Adamts6<br>Nek6<br>Csad<br>Zfp827<br>Irf7<br>Sorbs1<br>Gbp3<br>Atp10d<br>Obscn<br>Ppp1r15a							

Supplementary Table 2: Reagents

REAGENT	Source	Identifier	Concentration
**Antibodies**			
Biotin anti-TER-119 Clone:TER-119	ThermoFisher Scientific	13-5921-82	1:175
Biotin anti-Ly-6G/Ly-6C/Gr1 Clone:RB-8C5	ThermoFisher Scientific	13-5931-82	1: 175
Biotin anti-CD45R (B220) Clone:RA-6B2	ThermoFisher Scientific	13-0452-82	1:350
Biotin anti-CD11b Clone:M1/70	ThermoFisher Scientific	13-0112-82	1: 350
Biotin anti-CD3 Clone:17A2	ThermoFisher Scientific	13-0032-82	1:700
Biotin anti-CD4 Clone:RM4-5	ThermoFisher Scientific	13-0042-82	1: 700
Biotin anti-CD8a Clone:53-6.7	ThermoFisher Scientific	13-0081-82	1: 700
anti-clca3a1 Clone:10.01.01	DSHB	10.1.1	1:25
APC anti-mouse CD117 (c-kit)- Clone:2B8	Biolegend	105812	1:50
Streptavidin-eFluor450	ThermoFisher Scientific	48-4317-82	1:25
PE-Cy7 anti-mouse Ly-6A/E (Sca1) Clone:D7	ThermoFisher Scientific	25-5981-81	1:50
PE anti-mouse CD135 (Flt3)- Clone:A2F10	ThermoFisher Scientific	12-1351-82	1:100
FITC anti-mouse CD34 Clone:RAM34	ThermoFisher Scientific	11-0341-82	1:12
Brilliant Violet 711™ anti-Syrian Hamster (Secondary) Clone:G192-3	BD Bioscience	745460	1:100
PE anti-mouse CD45.1 Clone:A20	Biolegend	110708	1:100
PerCP/Cy5.5 anti-mouse CD45.2 Clone:104	Biolegend	109828	1:100
APC/Cy7 anti-mouse/human CD11b Clone:M1/70	Biolegend	101226	1:100
Brilliant Violet 605™ anti-mouse/human CD45R/B220 Clone:RA3-6B2	Biolegend	103243	1:100
Alexa Fluor® 700 anti-mouse CD4 Clone:L3T4, T4	Biolegend	100536	1:100
APC anti-mouse CD8a Clone:53-6.7	Biolegend	100712	1:100
PE/Cy7 anti-mouse Ly-6G/Ly-6C (Gr-1) Clone:RB6-8C5	Biolegend	108416	1:100
Alexa Fluor® 700 anti-mouse/human CD45R/B220 Clone:RA3-6B2	Biolegend	103232	1:100
APC anti-mouse CD3 Clone:17A2	Biolegend	100236	1:100
Alexa Fluor® 700 anti-mouse CD45.1 Clone:A20	Biolegend	110724	1:100
Brilliant Violet 785™ anti-CD45.2 Clone:104	Biolegend	109839	1:25
APC/Cy7 anti-mouse CD16/32 Clone:93	Biolegend	101328	1:100
Brilliant Violet 650™ anti-mouse CD127 Clone:A7R34	Biolegend	135043	1:50
TotalSeq™-A0203 anti-mouse CD150 (SLAM) Clone:TC15-12F12.2	Biolegend	115945	0,5 µg
TotalSeq™-A0911 anti-phycoerythrin (PE) Clone:PE001	Biolegend	408109	0,5 µg
TotalSeq™-A0429 anti-mouse CD48 Clone:HM48-1	Biolegend	103447	0,5 µg
TotalSeq™-A0238 Rat IgG 2α, λ Isotype control	Biolegend	400571	0,5 µg
TotalSeq™-A0241 Armenian Hamster IgG Isotype control	Biolegend	400973	0,5 µg
PE/Cy7 anti-Syrian hamster	Abcam	ab130807	1:50
FITC anti-mouse CD11b	ThermoFisher Scientific	11-0112-41	1:100

PE/Cy7 anti-mouse CD150	Biolegend	115913	1:50
Brilliant Violet 605™ anti-mouse CD127	Biolegend	135025	1:30
PE anti-CD34	Biolegend	152204	1:50
Anti-Vinculin (hVIN-1)	Sigma-Aldrich	#V9131	1:10000
Anti-Wwtr1 (V386)	Cell Signaling	#4883	1:1000
Anti-Yap1/Wwtr1	Santa Cruz	sc-101199	1:2000
Anti-PU.1/Spi1	Abcam	Ab227835	1:1000
Anti-GAPDH	Santa Cruz	Sc-365062	1:10000
Anti-mouse IgG-HRP	Santa Cruz	sc-2314	1:5000
Anti-rabbit IgG-HRP	Santa Cruz	sc-2313	1:5000
**Chemicals, Peptides, and Recombinant Proteins**			
StemSpan SFEM Medium	STEMCELL Technologies	09650	
IMDM (+GlutaMax)	ThermoFisher Scientific	31980030	
Recombinant mouse SCF	STEMCELL Technologies/PeproTech	78064.1/ AF-250-03	
Recombinant mouse TPO	STEMCELL Technologies/PeproTech	78072.1/ AF-315-14	
Recombinant mouse IL-3	STEMCELL Technologies	78042.1	
Recombinant mouse IL-6	STEMCELL Technologies	78052.1	
Human RetroNectin	Takara	T100B	
BD Pharm Lyse™ lysing Buffer	BD Biosciences	555899	
SYTOX Blue dead cell stain	ThermoFisher Scientific	S34857	
LIVE/DEAD™ Fixable Violet Dead Cell Stain Kit	ThermoFisher Scientific	L34964	
Fetal Bovine Serum	Gibco	#F7524-500ML	
**Critical Commercial Assays**			
innuMIX qPCR DSGreen Standard	Analytik Jena	845-AS-1300200	
RNeasy® Micro kit	Qiagen	74004	
NEBNext® Ultra RNA Library Prep kit for Illumina	NEB	E7530L	
Chromium Single Cell 3′ Library Construction Kit v3	10x Genomics	1000078	
Chromium Next GEM Single Cell ATAC Library & Gel Bead Kit v1.1	10x Genomics	1000176	
MinElute PCR Purification Kit	Qiagen	28006	
Agencourt AMPure XP	Beckman Coulter	A63881	
NEBNext® High-Fidelity 2X PCR Master Mix	NEB	M0541L	
ChIP DNA clean & Concentrator™	ZYMORESEARCH	D5201	
Passive Lysis Buffer	Promega	E194A	

Supplementary Table 3: Oligonucleotides

Primer / oligo	Sequence
**Sc-Seq**	
RPI1	CAAGCAGAAGACGGCATACGAGATCGTGATGTGACTGGAGTTCCTTGGCACCCGAGAATTCCA
RPI2	CAAGCAGAAGACGGCATACGAGATACATCGGTGACTGGAGTTCCTTGGCACCCGAGAATTCCA
10X Genomics SI	AATGATACGGCGACCACCGAGATCTACACTCTTTCCCTACACGACGCTC
**ATAC-Seq**	
V2_Ad1.1	AATGATACGGCGACCACCGAGATCTACACTAGATCGCTCGTCGGCAGCGTCAGATGTGTAT
V2_Ad1.2	AATGATACGGCGACCACCGAGATCTACACCTCTCTATTCGTCGGCAGCGTCAGATGTGTAT
V2_Ad1.3	AATGATACGGCGACCACCGAGATCTACACTATCCTCTTCGTCGGCAGCGTCAGATGTGTAT
V2_Ad1.4	AATGATACGGCGACCACCGAGATCTACACAGAGTAGATCGTCGGCAGCGTCAGATGTGTAT
V2_Ad1.5	AATGATACGGCGACCACCGAGATCTACACGTAAGGAGTCGTCGGCAGCGTCAGATGTGTAT
V2_Ad1.6	AATGATACGGCGACCACCGAGATCTACACACTGCATATCGTCGGCAGCGTCAGATGTGTAT
V2_Ad2.1	CAAGCAGAAGACGGCATACGAGATTCGCCTTAGTCTCGTGGGCTCGGAGATGTG
**qRT-PCR**	
mAmotl2_1_Fw	GGAGAAGAGTTGCCCACCTAT
mAmotl2_1_Rev	TCGAAGAGCTTCATCCTGTCG
mWwtr1_Fw	GAAGGTGATGAATCAGCCTCTG
mWwtr1_Rev	GTTCTGAGTCGGGTGGTTCTG
mb2M_1_Fw	AGCCGAACATACTGAACTGCTACG
mb2M_1_Rev	CGGCCATACTGTCATGCTTAACTC
Yap1_Fw	TGAGATCCCTGATGATGTACCAC
Yap1 Rev	TGTTGTTGTCTGATCGTTGTGAT
**shRNAs**	
shRen	TGCTGTTGACAGTGAGCGCAGGAATTATAATGCTTATCTATAGTGAAGCC ACAGATGTATAGATAAGCATTATAATTCCTATGCCTACTGCCTCGGA
shWwtr1#1	TGCTGTTGACAGTGAGCGCTGGTCACAATATGAATGATTATAGTGAAGCC ACAGATGTATAATCATTCATATTGTGACCAATGCCTACTGCCTCGGA
shWwtr1#2	TGCTGTTGACAGTGAGCGCACTTCTCAATTCTATATATAATAGTGAAGCC ACAGATGTATTATATATAGAATTGAGAAGTATGCCTACTGCCTCGGA
shPu1#1	TGCTGTTGACAGTGAGCGCGGCACCTTTTTGTATATTGAATAGTGAAGCCACAGATGTATTCAATATACAAAAA GGTGCCATGCCTACTGCCTCGGA
shPu1#2	TGCTGTTGACAGTGAGCGCCCACAACAACGAGTTTGAGAATAGTGAAGCCACAGATGTATTCTCAAACTCGTT GTTGTGGATGCCTACTGCCTCGGA
**Cloning**	
Wwtr1_Flag_EcoRI	GATCGAATTCATGGACTACAAAGACGATGACG
Wwtr1_Stop_NotI	GATCGCGGCCGCTTACAGCCAGGTTAGAAAGGGCT
MirE fw	TGAACTCGAGAAGGTATATTGCTGTTGACAGTGAGCG
MirE rev	TCTCGAATTCTAGCCCCTTGAAGTCCGAGGCAGTAGGC
