# Supplementary material for: Taz protects hematopoietic stem cells from an aging-dependent decrease in PU.1 activity
Source: Nat Commun. 2022 Sep 3;13:5187. doi: 10.1038/s41467-022-32970-1 (PMC9440927; doi:10.1038/s41467-022-32970-1)
Supplement: Supplementary file 2 — Reporting Summary [file 41467_2022_32970_MOESM2_ESM.pdf]

nature portfolio

Corresponding author(s): Björn von Eyss

Last updated by author(s): 2022/07/14

Reporting Summary

Nature Portfolio wishes to improve the reproducibility of the work that we publish. This form provides structure for consistency and transparency in reporting. For further information on Nature Portfolio policies, see our Editorial Policies and the Editorial Policy Checklist.

Statistics

For all statistical analyses, confirm that the following items are present in the figure legend, table legend, main text, or Methods section.

n/a	Confirmed	
☐	☒	The exact sample size (*n*) for each experimental group/condition, given as a discrete number and unit of measurement
☐	☒	A statement on whether measurements were taken from distinct samples or whether the same sample was measured repeatedly
☐	☒	The statistical test(s) used AND whether they are one- or two-sided<br>*Only common tests should be described solely by name; describe more complex techniques in the Methods section.*
☒	☐	A description of all covariates tested
☐	☒	A description of any assumptions or corrections, such as tests of normality and adjustment for multiple comparisons
☐	☒	A full description of the statistical parameters including central tendency (e.g. means) or other basic estimates (e.g. regression coefficient) AND variation (e.g. standard deviation) or associated estimates of uncertainty (e.g. confidence intervals)
☐	☒	For null hypothesis testing, the test statistic (e.g. *F*, *t*, *r*) with confidence intervals, effect sizes, degrees of freedom and *P* value noted<br>*Give P values as exact values whenever suitable.*
☒	☐	For Bayesian analysis, information on the choice of priors and Markov chain Monte Carlo settings
☒	☐	For hierarchical and complex designs, identification of the appropriate level for tests and full reporting of outcomes
☐	☒	Estimates of effect sizes (e.g. Cohen's *d*, Pearson's *r*), indicating how they were calculated

Our web collection on statistics for biologists contains articles on many of the points above.

Software and code

Policy information about availability of computer code

Data collection	BD FACSDiva 8.0.1
Data analysis	R (v4.1.0) r-project.org https://cran.r-project.org/bin/windows/base/<br>Ubuntu (v16.04) Ubuntu https://ubuntu.com<br>Samtools Samtools http://www.htslib.org<br>FastQC (v.0.11.5) Babraham Bioinformatics https://www.bioinformatics.babraham.ac.uk/projects/fastqc/<br>Bowtie2 (v2.2.9) Bowtie2 http://bowtie-bio.sourceforge.net/bowtie2/index.shtml<br>cutadapt (v2.10) Cutadapt https://cutadapt.readthedocs.io/en/stable/#<br>edgeR (v3.26.8) Bioconductor https://bioconductor.org/packages/release/bioc/html/edgeR.html<br>DESeq2 (v1.36.0) Bioconductor https://bioconductor.org/packages/release/bioc/html/DESeq2.html<br>AUCell (v1.6.1) Bioconductor https://bioconductor.org/packages/release/bioc/html/AUCell.html<br>chromVAR (v1.6.0) Bioconductor https://bioconductor.org/packages/release/bioc/html/chromVAR.html<br>HOMER Homer http://homer.ucsd.edu/homer/microarray/go.html<br>Genrich GitHub https://github.com/jsh58/Genrich<br>deepTools GitHub https://github.com/deeptools/deepTools/<br>CITE-seq-Count (v1.4.0) GitHub https://github.com/Hoohm/CITE-seq-Count<br>Cellranger (v3.1.0) 10x Genomics https://www.10xgenomics.com/<br>Cellranger-atac (v1.1.0) 10x Genomics https://www.10xgenomics.com/<br>ArchR (v1.0.1) https://www.archrproject.com/<br>Seurat (v4.0.3) Satija lab https://satijalab.org/seurat/<br>SEACR(v1.3) https://github.com/FredHutch/SEACR<br>nf-core ATAC pipeline(v1.2.1) https://nf-co.re/atacseq

FlowJo 9.9.6
FlowLogic 700.0 A

For manuscripts utilizing custom algorithms or software that are central to the research but not yet described in published literature, software must be made available to editors and reviewers. We strongly encourage code deposition in a community repository (e.g. GitHub). See the Nature Portfolio guidelines for submitting code & software for further information.

Data

Policy information about availability of data

All manuscripts must include a data availability statement. This statement should provide the following information, where applicable:

- Accession codes, unique identifiers, or web links for publicly available datasets
- A description of any restrictions on data availability
- For clinical datasets or third party data, please ensure that the statement adheres to our policy

All data generated by Next-generation sequencing were uploaded to the GEO repository with the reference series GSE157464.

Field-specific reporting

Please select the one below that is the best fit for your research. If you are not sure, read the appropriate sections before making your selection.

[x] Life sciences [ ] Behavioural & social sciences [ ] Ecological, evolutionary & environmental sciences

For a reference copy of the document with all sections, see nature.com/documents/nr-reporting-summary-flat.pdf

Life sciences study design

All studies must disclose on these points even when the disclosure is negative.

Sample size	Sample sizes for animal experiments were determined based on previous experiments analyzing similar biological aspects (PMID: 22385964).
Data exclusions	Data for transplantation studies included an outlier analysis using the R 'outliers' package. This led to an exclusion of one data point in Fig. 6e T cells for shTAZ#2.
Replication	All data are derived from biological replicates (different mice). All transplantation data are derived from at least two, mostly three, different rounds of transplantation. All replicates yielded similar results.
Randomization	The animals were randomly selected to be assigned to an experimental vs. control group.
Blinding	Blinding was not performed since this was logistically not possible: all mice need to be assigned to a dedicated group (experimental vs. control) in our mouse database. Thus, the people performing experiments will inevitably know which mouse received which treatment.

Reporting for specific materials, systems and methods

We require information from authors about some types of materials, experimental systems and methods used in many studies. Here, indicate whether each material, system or method listed is relevant to your study. If you are not sure if a list item applies to your research, read the appropriate section before selecting a response.

Materials & experimental systems

n/a	Involved in the study	
[ ]	[x]	Antibodies
[ ]	[x]	Eukaryotic cell lines
[x]	[ ]	Palaeontology and archaeology
[ ]	[x]	Animals and other organisms
[x]	[ ]	Human research participants
[x]	[ ]	Clinical data
[x]	[ ]	Dual use research of concern

Methods

n/a	Involved in the study	
[x]	[ ]	ChIP-seq
[ ]	[x]	Flow cytometry
[x]	[ ]	MRI-based neuroimaging

Antibodies

Antibodies used	anti-TER-119 Clone:TER-119 ThermoFisher Scientific 13-5921-82<br>anti-Ly-6G/Ly-6C/Gr1 Clone:RB-8C5 ThermoFisher Scientific 13-5931-82<br>anti-CD45R (B220) Clone:RA-6B2 ThermoFisher Scientific 13-0452-82<br>anti-CD11b Clone:M1/70 ThermoFisher Scientific 13-0112-82

anti-CD3 Clone:17A2 ThermoFisher Scientific 13-0032-82
anti-CD4 Clone:RM4-5 ThermoFisher Scientific 13-0042-82
anti-CD8a Clone:53-6.7 ThermoFisher Scientific 13-0081-82
anti-clca3a1 Clone:10.01.01 DSHB 10.1.1
APC anti-mouse CD117 (c-kit)- Clone:2B8 Biolegend 105812
Streptavidin-eFluor450 Clone: ThermoFisher Scientific 17-4317-82
PE-Cy7 anti-mouse Ly-6A/E (Sca1) Clone:D7 ThermoFisher Scientific 25-5981-81
PE anti-mouse CD135 (Flt3)- Clone:A2F10 ThermoFisher Scientific 12-1351-82
FITC anti-mouse CD34 Clone:RAM34 ThermoFisher Scientific 11-0341-82
Brilliant Violet 711™ anti-Syrian Hamster (Secondary) Clone:G192-3 BD Bioscience 745460
PE anti-mouse CD45.1 Clone:A20 Biolegend 110708
PerCP/Cy5.5 anti-mouse CD45.2 Clone:104 Biolegend 109828
APC/Cy7 anti-mouse/human CD11b Clone:M1/70 Biolegend 101226
Brilliant Violet 605™ anti-mouse/human CD45R/B220 Clone:RA3-6B2 Biolegend 103243
Alexa Fluor® 700 anti-mouse CD4 Clone:L3T4, T4 Biolegend 100536
APC anti-mouse CD8a Clone:53-6.7 Biolegend 100712
PE/Cy7 anti-mouse Ly-6G/Ly-6C (Gr-1) Clone:RB6-8C5 Biolegend 108416
Alexa Fluor® 700 anti-mouse/human CD45R/B220 Clone:RA3-6B2 Biolegend 103232
APC anti-mouse CD3 Clone:17A2 Biolegend 100236
Alexa Fluor® 700 anti-mouse CD45.1 Clone:A20 Biolegend 110724
Brilliant Violet 785™ anti-CD45.2 Clone:104 Biolegend 109839
APC/Cy7 anti-mouse CD16/32 Clone:93 Biolegend 101328
Brilliant Violet 650™ anti-mouse CD127 Clone:A7R34 Biolegend 135043
TotalSeq™-A0203 anti-mouse CD150 (SLAM) Clone:TC15-12F12.2 Biolegend 115945
TotalSeq™-A0911 anti-phycoerythrin (PE) Clone:PE001 Biolegend 408109
TotalSeq™-A0429 anti-mouse CD48 Clone:HM48-1 Biolegend 103447
PE/Cy7 anti-Syrian hamster Abcam ab130807
FITC anti-mouse CD11b ThermoFisher Scientific 11-0112-41
PE/Cy7 anti-mouse CD150 Biolegend 115913
Brilliant Violet 605™ anti-mouse CD127 Biolegend 135025
PE anti-CD34 Biolegend 152204
PE Ki-67 BD Pharmingen 556027
Vinculin (hVIN-1) Sigma-Aldrich #V9131
Wwtr1 (V386) Cell Signaling #4883
Yap1/Wwtr1 Santa Cruz sc-101199
Anti-mouse IgG-HRP Santa Cruz sc-2314
Anti-rabbit IgG-HRP Santa Cruz sc-2313

Validation

The Clca3a1 antibody was validated by matching its signal with Clca3a1 mRNA expression in the different populations: LT-HSC, ST-HSC and MPP (see also Fig. 2c-e and Supplementary Figure 1).
The other antibodies are all well-established commercially available monoclonal antibodies and were not further validated.

Flow cytometry antibodies were validated by the manufacturer and were used in various publications. Statements of antibody validation can be found on the manufacturer websites along with the relevant references.

Western blot antibodies were tested by the manufacturers in various cell lines, detailed information and western blot pictures are provided on the manufacturers websites:
- Anti-hVIN-1 antibody (#V9131, Sigma Aldrich) was tested in HeLa, COS7, NIH-3T3, RAT2, CHO, MDBK and MDCK cell lines.
- Anti-Wwtr1 (#4883, cell signalling) was tested in HeLa, mouse ESC, NCCIT, F9, E6-5, A431, NIH/3T3, PC12 cells.
- Anti-Yap1/Wwtr1 (sc-101199, Santa Cruz) tested in 293T, Hela and PC-3 cells.
- Anti-PU.1/Spi1 antibody (Ab227835, Abcam) was used to immunoprecipitated Spi1 from J774A.1 (mouse reticulum cell sarcoma monocyte macrophage) whole cell lysate at 1/30 dilution (2ug in 0.35mg lysates). Western blot was performed on the immunoprecipitate at 1:1000 dilution.
-Anti-GAPDH antibody (Sc-365062, Santa Cruz) Western Blot of GAPDH expression in HeLa, Jurkat, MCF7, A-431 and HL-60 whole cell lysates.

All TotalSeq antibodies (Biolegend) were quality control tested by the manufacturer in immunofluorescent staining and with flow cytometric analysis and the oligomer sequence was confirmed by sequencing.

Eukaryotic cell lines

Policy information about cell lines

Cell line source(s)

Takara (LentiX cells for lentivirus production)
Leif Carlson, Umea University, Sweden (BM-HPC#5 cells)

Authentication	The cell lines were not authenticated.
Mycoplasma contamination	All cell lines were tested negative for mycoplasma contamination.
Commonly misidentified lines (See ICLAC register)	NA

Animals and other organisms

Policy information about studies involving animals; ARRIVE guidelines recommended for reporting animal research

Laboratory animals	CD45.2: C57BL/6JRj: Sex: female; age; young(3-5 months) old (22-28 months)<br>CD45.1: LY5.1 (B6.SJL-PtprcaPepcb/BoyCrl): Sex: female; age:8-12 weeks<br>CD45.1/2: C57BL/6JRj / B6.SJL-PtprcaPepcb/BoyCrl F1: Sex: female; age: 10-20 weeks
Wild animals	This study did not involve wild animals
Field-collected samples	This study did not involve samples collected from the field
Ethics oversight	All animal experiments were performed according to protocols approved by the state government of Thuringia under the animal experiment licenses FLI-17-024 and FLI-19-012.

Note that full information on the approval of the study protocol must also be provided in the manuscript.

Flow Cytometry

Plots

Confirm that:

- [x] The axis labels state the marker and fluorochrome used (e.g. CD4-FITC).
- [x] The axis scales are clearly visible. Include numbers along axes only for bottom left plot of group (a 'group' is an analysis of identical markers).
- [x] All plots are contour plots with outliers or pseudocolor plots.
- [x] A numerical value for number of cells or percentage (with statistics) is provided.

Methodology

Sample preparation	Isolation of primary HSPCs from mouse and flow cytometry and c-Kit enrichment:<br>Femurs, tibias, hips, humeri and spine were isolated from female C57BL/6JRj mice (Janvier Labs) of various ages and crushed in ice-cold FCM buffer (2% FCS, 1 mM EDTA in PBS). Cells were filtered through a 70 µm cell strainer and subjected to c-kit enrichment using an anti-c-Kit-APC antibody (BioLegend), anti-APC magnetic beads (Miltenyi Biotec) and LS MACS columns (Miltenyi Biotec). c-kit enriched cells were then used for further stainings.<br><br>Peripheral blood analysis:<br>Blood samples were collected from the Vena facialis into tubes containing 8 µl of 0.5 M EDTA and stained with fluorophore-conjugated antibodies. Red blood cells were then lysed in 1X BD Pharm Lyse lysing solution (BD Biosciences) for 8-10 min. Cells were washed twice in FCM, resuspended in fresh FCM and filtered through a 40 µm mesh.<br><br>Bone marrow analysis:<br>Femurs, tibias, hips, humeri and spine were isolated from female C57BL/6JRj mice (Janvier Labs) of various ages and crushed in ice-cold FCM buffer (2% FCS, 1 mM EDTA in PBS). Cells were filtered through a 70 µm cell strainer and stained with fluorophore-conjugated antibodies.
Instrument	Sorting:<br>BD FACSAria Fusion cell sorter<br>BD FACS Melody<br><br>Analysis:<br>BD LSR Fortessa
Software	BD FACSDiva 8.0.1<br>FlowJo 9.9.6<br>FlowLogic 700.0 A
Cell population abundance	We determined post-sort purities when pooled samples were collected, except for RNA isolation as samples were directly sorted into lysis buffer. For reanalysis, the sorted samples were removed from the collection tubes for their intended use.

Afterwards, the collections tubes were briefly rinsed with a small volume of FCM buffer and used for reanalysis.

Gating strategy	The gating strategies are given as Supplementary Figures. To identify dead cells in the FSC/SSC population SytoxBlue was added to all samples and based on this, the FSC/SSC was set to exclude dead cells. Isotype controls and/or fluorescence-minus-one (FMO) controls were used to define negative cell populations.

☒ Tick this box to confirm that a figure exemplifying the gating strategy is provided in the Supplementary Information.
